# Suspension survival mediated by PP2A-STAT3-Col XVII determines tumour initiation and metastasis in cancer stem cells

**DOI:** 10.1038/ncomms11798

**Published:** 2016-06-16

**Authors:** Chen-Chi Liu, Shih-Pei Lin, Han-Shui Hsu, Shung-Haur Yang, Chiu-Hua Lin, Muh-Hwa Yang, Mien-Chie Hung, Shih-Chieh Hung

**Affiliations:** 1 Institute of Clinical Medicine, National Yang-Ming University, Taipei 112, Taiwan; 2 Institute of Emergency and Critical Care Medicine, School of Medicine, National Yang-Ming University, Taipei 112, Taiwan; 3 Department of Surgery, Taipei Veterans General Hospital, Taipei 112, Taiwan; 4 Department of Surgery, School of Medicine, National Yang-Ming University, Taipei 112, Taiwan; 5 Department of Oncology, Taipei Veterans General Hospital, Taipei 112, Taiwan; 6 Graduate Institute of Cancer Biology, College of Medicine, Center for Molecular Medicine, China Medical University and Hospital, Taichung 404, Taiwan; 7 Department of Molecular and Cellular Oncology, The University of Texas M.D. Anderson Cancer Center, Houston, Texas 77030, USA; 8 Department of Medical Research and Education, Stem Cell Laboratory, Taipei Veterans General Hospital, Taipei 112, Taiwan; 9 Institute of Biomedical Sciences, Academia Sinica, Taipei 11529, Taiwan; 10 Department of Orthopedics, Integrative Stem Cell Center, China Medical University Hospital, Taichung 404, Taiwan; 11 Graduate Institute of Clinical Medical Science, China Medical University, Taichung 404, Taiwan

## Abstract

Targeting tumour-initiating cells (TICs) would lead to new therapies to cure cancer. We previously demonstrated that TICs have the capacity to survive under suspension conditions, while other cells undergo anoikis. Here we show that TICs exhibit increased phosphorylation levels of S727STAT3 because of PP2A inactivation. Collagen 17 gene expression is upregulated in a STAT3-dependent manner, which also stabilizes laminin 5 and engages cells to form hemidesmosome-like junctions in response. Blocking the PP2A-S727STAT3-collagen 17 pathway inhibits the suspension survival of TICs and their ability to form tumours in mice, while activation of the same pathway increases the suspension survival and tumour-initiation capacities of bulk cancer cells. The S727STAT3 phosphorylation levels correlate with collagen 17 expression in colon tumour samples, and correlate inversely with survival. Finally, this signalling axis enhances the ability of TIC to form tumours in mouse models of malignant lung cancer pleural effusion and spontaneous colon cancer metastasis.

## Abstract (web summary)

Tumour initiating cells (TICs) are anoikis resistant in suspension culture and they are critical for initiating tumorigenesis *in vivo*. Here, the authors show that these features are promoted by hemidesmosome-like structures enriched in laminin V and collagen XVII 5 upregulated in TICs by phospho-STAT3 whose levels are increased through PP2A inactivation.

## Graphical abstract

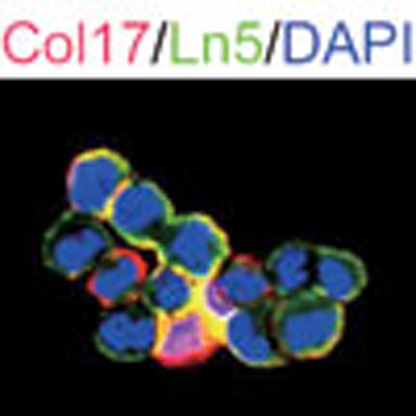

The ‘Seed and Soil' theory concludes that cancer cells (‘seeds') could only grow in congenial conditions (‘soil')^1^. The soil, now referred to as the niche of cancer cells, is composed of extracellular matrices (ECM) and cellular components in the microenvironment^2^. Recently, cancer cells have been found to carry their ECM during the metastasis processes^3^. Moreover, cancer cells when delivered in matrigel, a mixture of ECM, also increase the ability to initiate tumour formation^4^. These data highly suggest that the original ECM around cancer cells are important for their survival and growth at the metastasis and tumour-initiation microenvironments, which at most are characterized as suspension conditions.

Tumour-initiating cells (TICs) or cancer stem cells are subpopulations of cancer cells responsible for tumour initiation, metastasis and treatment resistance^5^,^6^. Highly pure populations of TICs have been obtained by spheroid condition, a suspension culture in a serum-free medium^7^. Cancer cells proliferate/differentiate under anchorage-independent conditions, giving rise to clonal spheroids, which can in part recapitulate the primary tumour expression profile^8^. Although previous data strongly implicate that TICs or normal stem cells may have better suspension-survival ability than other cells^9^,^10^,^11^, there are few, if any, studies investigating specifically whether these cells increased in suspension-survival ability, and elucidating the underlying mechanisms.

In the current study, we found TICs, the seeds, produce their own soil, thereby increasing the capacity for suspension survival at the metastasis and tumour-initiation microenvironments. We examined whether TICs, from colorectal cancer samples and cell lines and other cancer cell lines from the lung, brain and breast cancers, increased the ability to survive under various suspension conditions both *in vitro* and *in vivo*. Moreover, we investigated the underlying mechanism that TICs mediated to increase the suspension-survival ability. More importantly, we investigated the correlation of the related signalling pathways with the tumour staging and survival of patients in human colorectal cancer. Finally, we demonstrated the relevant role of this TIC characteristic and the same pathway in inducing spontaneous colorectal cancer metastasis and malignant lung cancer pleural effusion. Our data show that collagen XVII-laminin5 upregulated by PP2A-^S727^STAT3 mediates anoikis resistance, which plays an important role in the determination of tumour initiation and metastasis in cancer stem cells.

## Results

### TICs show suspension-survival ability

**Figure 1:**
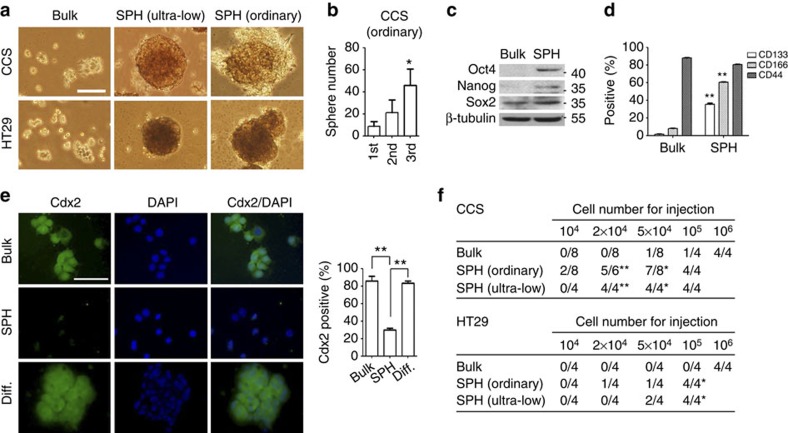
Characterization of spheroid culture-enriched TICs. (**a**) Representative pictures of sphere formed from CCS and HT29 in spheroid culture under non-adherent (ultra-low dish) or adherent (ordinary) condition. Scale bar, 50 μm. (**b**) CCS cancer cells in spheroid culture were subcultured for every 15 days. Quantification of number of CCS spheres for three consequent generations with size over 250 μm for five pictures. (**c**) Western blot and (**d**) flow cytometry analysis for bulk cancer cells (Bulk) and spheroid culture-enriched TICs (SPH). (**e**) Left, immunostaining for Cdx2 of bulk cancer cells, spheroid culture-enriched TICs and TICs after differentiation (Diff.) in matrigel for 1 month. Scale bar, 50 μm. Right, quantification of positive cells was performed with five pictures per sample. (**f**) Tumorigenicity of bulk cancer cells and spheroid-enriched TICs derived from CCS and HT29 cells. The stem cell frequencices for CCS bulk and sphere culture (ultra-low) are 1:35,000 and 1:16,000, respectively, and for HT29 bulk and sphere culture (ultra-low) are 1:530,000 and 73,000, respectively. The results are expressed as mean±s.d. of three independent experiments. Asterisks indicate significant differences (*P<0.05, **P<0.01 versus first or bulk) determined by one-way analysis of variance (ANOVA; **b**,**e**), Student's *t*-test (**d**) and Fisher's exact test (**f**).

To avoid the unreliability of the isolation of TICs by putative surface markers and the selection of cells preferred to survive suspension in ultra-low dishes, TICs were enriched by the spheroid culture both in ultra-low (Fig. 1a) and ordinary plastic dishes (Fig. 1a) from primary cancer cells, CCS, or cell line, HT29. Enriched TICs increased in the ability to form secondary and tertiary spheres (Fig. 1b), express pluripotency factors (Fig. 1c), increase TIC surface makers^12^, such as CD133 and CD166 (Fig. 1d), decrease colorectal differentiation marker^12^, such as Cdx2 (Fig. 1e), differentiate when reseeded with serum in Matrigel (Fig. 1e) and generate tumours upon inoculation underneath the skin of immunodeficient mice (Fig. 1f).

**Figure 2:**
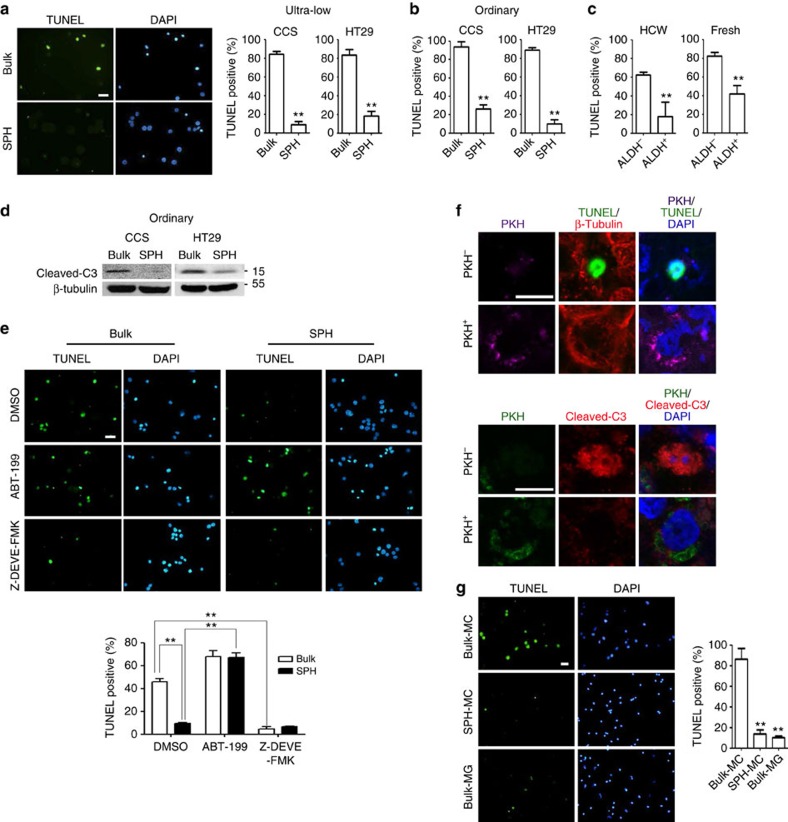
Spheroid culture-enriched TICs show better suspension-survival ability. (**a**) Left, representative pictures of TUNEL staining for HT29. Right, quantification of TUNEL-positive cells. (**b**,**d**) Bulk cancer cells (Bulk) and spheroid culture-enriched TICs (SPH) were cultured under spheroid condition for (**b**) 24 h and (**d**) 10 h, followed by (**b**) TUNEL assay and (**d**) western blot analysis, respectively. (**c**) ALDH^−^ and ALDH^+^cells isolated from HCW primary liver metastasis colorectal cancer cells were cultured under spheroid condition for 24 h, followed by TUNEL assay. (**e**) HT29 bulk cancer cells and spheroid culture-enriched TICs were cultured under spheroid condition in the presence of Bcl-2 inhibitor (ABT-199, 1 μM), caspase 3 inhibitor (Z-DEVE-FMK, 10 μM) and dimethylsulphoxide (vehicle control) for 24 h, followed by TUNEL assay. Top, representative pictures of TUNEL staining. Bottom, quantification of TUNEL-positive cells. (**f**) CCS cancer cells were labelled with PKH, followed by culture under spheroid condition for 15 days. Spheroids were subjected to TUNEL staining and immunostaining for β-tubulin and cleaved-caspase3 (C3). (**g**) CCS bulk cancer cells and spheroid culture-enriched TICs were subjected to *in vivo* suspension condition in the absence or presence of 5% matrigel (MG) for 24 h. Left, representative pictures of TUNEL staining. Right, quantification of TUNEL-positive cells. Quantification of TUNEL assay was performed with five pictures for each sample. The results are expressed as mean±s.d. of three independent experiments. Asterisks indicate significant differences (**P<0.01) determined by Student's *t*-test and one-way ANOVA. Scale bar, 20 μm.

The enriched TICs of CCS and HT29 showed less apoptosis than their bulk cancer cells at 24 h of suspension with serum deprivation as evident by TdT-mediated dUTP nick end labelling (TUNEL; Fig. 2a,b) as well as Annexin V/PI (propidium iodide) assay (Supplementary Fig. 1a), independent of culture dishes used to enrich TICs. Moreover, TICs selected using positive aldehyde dehydrogenase (ALDH) activity^13^ from primary liver metastasis of colorectal cancer cells HCW^12^ and fresh colorectal cancer specimens also showed superior suspension survival than ALDH^−^ cancer cells (Fig. 2c and Supplementary Fig. 1b,c). Consistent with previous findings^14^, our results also showed that the levels of cleaved caspase 3 were greater in bulk cells than in enriched TICs (Fig. 2d). Moreover, we also demonstrate that activation of caspase 3 or inhibition of Bcl-2 played a major role in mediating suspension-induced cell death (Fig. 2e). Increased suspension survival by TICs was also demonstrated in condition with serum using poly-HEMA-coated dishes^15^ (Supplementary Fig. 2a) and in long-term soft-agar culture (Supplementary Fig. 2b). Moreover, the increase of suspension survival in TICs from CCS was attributed mainly to CD133^+^, rather than to CD133^−^ cells (Supplementary Fig. 2c). When compared the survival of TICs with bulk cancer cells using the mitotic-quiescence properties of TICs to retain specific dyes, such as PKH26, in culture^16^, PKH-positive cells survived in spheres compared with PKH-negative cells at 15 days of CCS spheroid culture, which were positive for TUNEL and cleaved caspase 3 (Fig. 2f).

To demonstrate that TICs also increased suspension survival *in vivo*, we subcutaneously implanted nude mice with silica tubes containing methylcellulose-delivered cells. Transplants recovered 24 h later revealed the survival of the majority of enriched TICs from CCS, while bulk cancer cells underwent apoptosis, which can be prevented by adding ECM in methylcellulose (Fig. 2j). These data together suggest that TICs display better suspension survival under various conditions, both *in vitro* and *in vivo*.

### Phosphorylation of S727STAT3 mediates suspension survival

**Figure 3:**
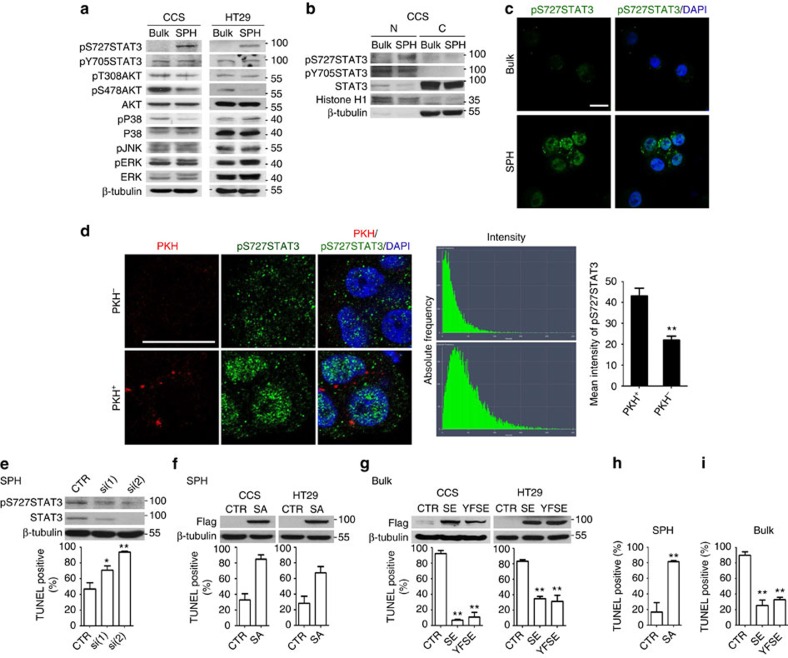
Activation of STAT3 at S727 mediates the suspension-survival ability of spheroid-enriched TICs. (**a**–**c**) Bulk cancer cells (Bulk) and spheroid culture-enriched TICs (SPH) were cultured under spheroid condition for 10 h, followed by (**a**) western blot assay of total protein or (**b**) fractionated protein (N, nucleus and C, cytoplasm) and (**c**) immunostaining followed by examination with a confocal microscope (CCS). (**d**) HT29 cancer cells were labelled with PKH, followed by culture under spheroid condition for 15 days. Spheroids were subjected to immunostaining followed by examination with a confocal microscope, and quantification of fluorescence intensity with ZEISS microscope software ZAN. (**e**) Spheroid cultures of CCS overexpressed with control shRNAs (CTR) or different STAT3 shRNAs, si(1) and si(2), were suspended and cultured under spheroid condition for 24 h, followed by TUNEL assay. Top, western blot analysis of STAT3 knockdown efficiency. Bottom, quantification of TUNEL-positive cells. (**f**,**h**) Spheroid culture cells overexpressed with control plasmids (CTR) or S727A point-mutated STAT3 (SA). (**g**,**i**) Bulk cancer cells overexpressed with control plasmids (CTR) or S727E point-mutated STAT3 (SE) without or with Y705F mutation (YFSE). (**f**,**g**) Cells were cultured under spheroid condition for 24 h, followed by TUNEL assay. Top, western blot analysis. Bottom, quantification of TUNEL-positive cells. (**h**,**i**) Cells derived from CCS were subjected to *in vivo* suspension condition for 24 h, followed by TUNEL assay. The results are expressed as mean±s.d. of three independent experiments. Asterisks indicate significant differences (*P<0.05, **P<0.01 versus PKH+ or CTR) determined by Student's *t*-test and one-way ANOVA. Scale bar, 20 μm.

Anoikis is apoptosis-induced by the loss of cell adhesion, and is associated with the state of cell differentiation^17^. Immunoblotting analysis revealed reduced phospho-^S473^AKT levels in TICs compared with bulk cancer cells and no obvious differences in the activation of other anoikis-related pathways^14^ between TICs and bulk cancer cells under suspension (Fig. 3a), suggesting that these pathways are not responsible for suspension survival in TICs. We and others have demonstrated that the activation of the JAK2-^Y705^STAT3 pathway by IL-6 leads to an increased tumour initiation ability in colorectal, lung and breast TICs^12^,^18^,^19^. Phosphorylation of STAT3 was increased at S727 in TICs compared with bulk cancer cells, while there was no difference between phosphorylation at Y705 (Fig. 3a,b), between TICs and bulk cancer cells (Fig. 3a). Moreover, phosphorylated S727 was located in the nucleus, where STAT3 transcription factor functions^20^ (Fig. 3b,c). Consistently, S727 phosphorylation was also increased in PKH-positive cells under long-term spheroid culture (Fig. 3d). Moreover, STAT3 knockdown (Fig. 3e) or overexpression of S727A point-mutated STAT3, an inactive form, increased apoptosis in TICs (Fig. 3f), while overexpression of S727E point-mutated STAT3, an active form, decreased apoptosis in bulk cancer cells (Fig. 3g). Similar results were also found in *in vivo* studies (Fig. 3h,i). However, STAT3 knockdown or overexpression of both mutated STAT3s did not have any effects on the survival and apoptosis in bulk cancer in monolayer culture (Supplementary Fig. 2d). More importantly, phosphorylation of STAT3 at Y705 was dispensable for the inhibition of apoptosis in bulk cancer cells by overexpression with S727E point-mutated STAT3 (Fig. 3g,i), suggesting that the phosphorylation of ^S727^STAT3 mediates suspension survival in TICs.

### Col XVII serves as a downstream target of phosphorylated S727STAT3

**Figure 4:**
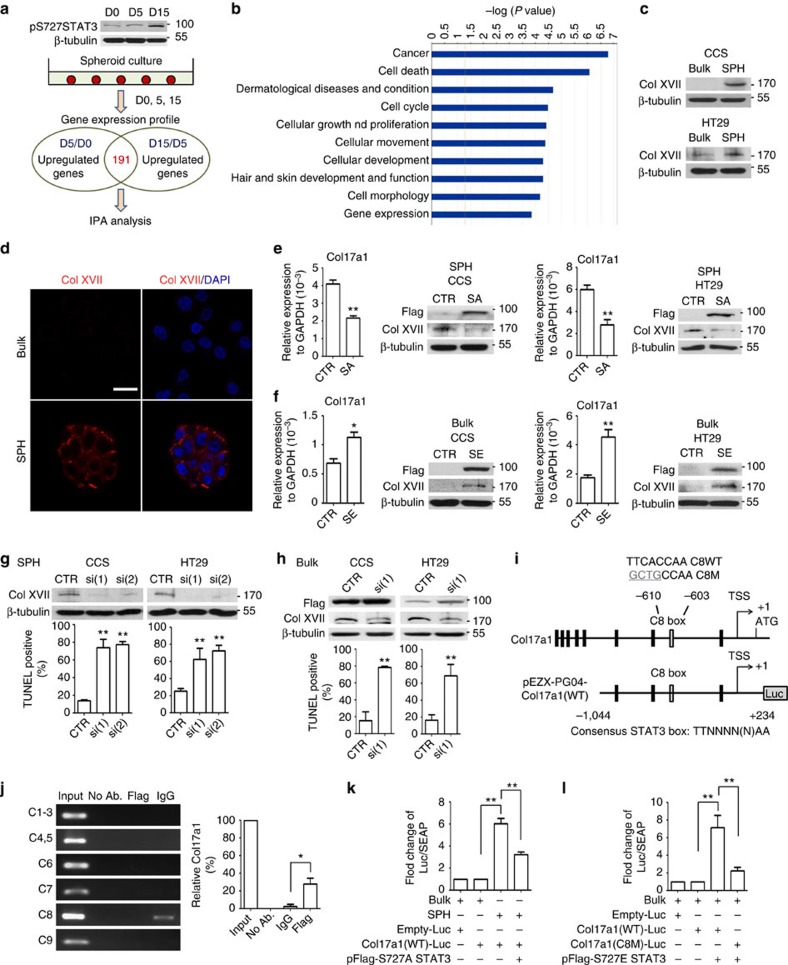
Col XVII upregulated by S727-phosphorylated STAT3 mediates the suspension-survival ability of enriched TICs. (**a**) Top, western blot for spheroid culture of days 0, 5 and 15. Bottom, the schematic representation of CCS cell preparation for investigating gene expression profiles and subsequent data analysis. (**b**) Upregulated genes in CCS-derived TICs were distributed in cancer and cell death categories through Gene Ontology (GO) and pathway analysis. (**c**–**d**) Bulk cancer cells (Bulk) and spheroid culture-enriched TICs (SPH) were cultured under spheroid condition for 10 h, followed by (**c**) western blot, and (**d**) immunostaining followed by examination with a confocal microscope (CCS). (**e**) Cells overexpressed with control plasmids (CTR) or S727A point-mutated STAT3 (SA) were assayed for mRNA and protein levels. (**f**) Cells overexpressed with control plasmids (CTR) or S727E point-mutated STAT3 (SE) were assayed for mRNA and protein levels. (**g**) Spheroid cultures or (**h**) bulk cancer cells expressing S727E point-mutated STAT3 were overexpressed with control shRNAs (CTR) or different Col17a1 shRNAs, si(1) and si(2). Cells were cultured under spheroid condition for 24 h, followed by TUNEL assay. Top, western blot of Col17a1 knockdown efficiency. Bottom, quantification of TUNEL-positive cells. (**i**) Genomic organization of the region flanking the promoter region of Col17a1 (top panel) and the schematic representation of the pEZX-PG04-Col17a1 reporter construct. Transcription start site, TSS. (**j**) Left, ChIP analysis of bulk cancer cells expressing S727E point-mutated STAT3. The chromatin was incubated either without antibodies, with an anti-FLAG antibody or with an isotype IgG antibody. Fragments of the eighth binding site in the Col17a1 promoter (C8) were amplified by PCR. Right, quantification of DNA binding with quantitative RT–PCR. Input, 4% of total input lysate. (**k**) Spheroid culture-enriched TICs (SPH) expressing S727A point-mutated STAT3 were employed to analyse the role of S727 phosphorylation in activating the Col17a1 promoter. (**l**) Mutational analysis of the eighth binding site in the Col17a1 promoter. Reporter constructs containing WT and box 8 mutations were employed to analyse the importance of this site in mediating activation by STAT3. The results are mean±s.d. of three independent experiments. Asterisks indicate significant differences (*P<0.05, **P<0.01 versus CTR) determined by Student's *t*-test and one-way ANOVA. Scale bar, 20 μm.

Through Gene Ontology analysis of genes upregulated along with the increase in spheroid culture time (Fig. 4a), we observed that the genes involved in spheroid culture were specifically enriched in the highly expressed categories, such as cancer and cell death categories (Fig. 4b). Surprisingly, the most upregulated gene in spheroid culture was *Col17a1* (Supplementary Table 1), which has not been reported to be involved in tumorigenicity and survival ability of cancer cells. We first confirmed reliability of the microarray data (Supplementary Table 1 and Supplementary Fig. 3a) and the upregulation of Col17a1 during spheroid culture using quantitative reverse transcriptase PCR (RT–PCR), immunoblotting and immunofluorescence (Supplementary Figs 3b and 4c,d). We then demonstrated that overexpression of S727A point-mutated STAT3 reduced Col17a1 expression in TICs (Fig. 4e), while overexpression of S727E point-mutated STAT3 increased Col17a1 expression in bulk cancer cells (Fig. 4f). Most interestingly, knockdown of Col17a1 reduced suspension survival in enriched TICs (Fig. 4g) and in bulk cancer cells expressing S727E point-mutated STAT3 (Fig. 4h).

Chromatin immunoprecipitation (ChIP) assay of the Col17a1 promoter (Fig. 4i) revealed that fragments containing the eighth putative STAT3-binding sites, TTNNNN(N)AA (−610 ∼ −603), but not other binding sites, were immunoprecipitated with anti-Flag antibody in HT29 expressing S727E point-mutated STAT3 (Fig. 4j). Moreover, the promoter luciferase reporter assay showed that Col17a1 promoter activity was greater in enriched TICs than in bulk cancer cells (Fig. 4k). We further demonstrated that transfection of S727A point-mutated STAT3 inactivated the Col17a1 promoter in enriched TICs (Fig. 4k), and transfection of S727E point-mutated STAT3 activated the wild-type (WT) Col17a1 promoter activity but not the eighth binding site-mutated Col17a1 promoter in bulk cancer cells (Fig. 4l). These data together suggest that Col17a1 plays an essential role in suspension survival mediated by S727-activated STAT3 in TICs.

### Suspension survival mediated by Col XVII requires laminin 5

**Figure 5:**
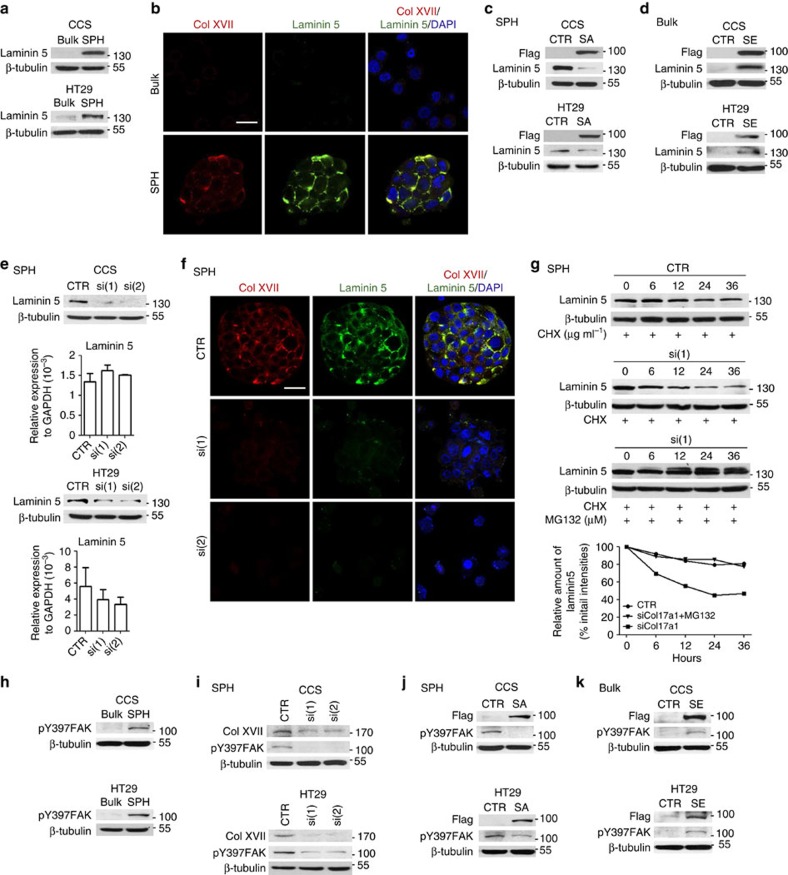
Col XVII-mediated suspension survival depends on laminin 5 and FAK activation. (**a**,**b**) Bulk cancer cells (Bulk) and spheroid culture-enriched TICs (SPH) were cultured in spheroid condition for 10 h, followed by (**a**) western blot assay and (**b**) immunofluorescence followed by examination with a confocal microscope. (**c**) Spheroid culture of cells overexpressed with control plasmids (CTR) or S727A point-mutated STAT3 (SA) were assayed for protein levels. (**d**) Bulk cancer cells overexpressed with control plasmids (CTR) or S727E point-mutated STAT3 (SE) were assayed for protein levels. (**e**,**f**) Spheroid cultures of cells overexpressed with control shRNAs (CTR) or different Col17a1 shRNAs, si(1) and si(2), were assayed for (**e**) mRNA and protein levels, and (**f**) immunostaining (CCS) followed by examination with a confocal microscope. (**g**) Top, western blot analysis for spheroid culture-enriched TICs expressing control shRNAs (CTR) or Col17a1 shRNAs, si(1) or si(2), treated with 50 μg ml^−1^ cycloheximide in the presence or absence of 10 μM MG132 for 6, 12, 24 and 36 h. Bottom, graphical representation of the relative band intensities of laminin 5 protein. The percentage of initial intensities of laminin 5 protein in cell extracts normalized with respect to β-tubulin amounts was plotted against time. (**h**) Bulk cancer cells and spheroid culture-enriched TICs were cultured under spheroid condition for 10 h, followed by western blot assay. (**i**) Western blot analysis for spheroid culture-enriched TICs expressing control shRNAs (CTR) or Col17a1 shRNAs, si(1) or si(2). (**j**) TICs (SPH) overexpressed with control plasmids (CTR), S727A point-mutated STAT3 (SA) were assayed for protein levels. (**k**) Bulk cells overexpressed with control plasmids (CTR), S727E point-mutated STAT3 (SE) were assayed for protein levels. Scale bar, 20 μm.

Previous report demonstrates that Col XVII and laminin 5 are components of hemidesmosome for mediating cell adhesion^21^. Moreover, laminin 5 was reported to regulate anchorage-independent survival through downstream signalling, such as FAK^22^. We showed an increase in the protein level of laminin 5 in TICs but not in bulk cancer cells (Fig. 5a) and the colocalization of Col XVII and laminin 5 in the membrane and cytoplasm of TICs (Fig. 5b). We further showed reduction of the laminin 5 protein level in spheroid culture of cells that were overexpressed with S727A point-mutated STAT3 (Fig. 5c), but increment of laminin 5 protein level in bulk cancer cells that were overexpressed with S727E point-mutated STAT3 (Fig. 5d). Moreover, knockdown of Col17a1 in TICs reduced the protein level but not the mRNA level of laminin 5 (Fig. 5e,f). Interestingly, the decrease in laminin 5 protein level was because of proteasome degradation, which was restored by treatment with proteasome inhibitor, MG132 (Fig. 5g). Moreover, FAK, a downstream mediator of laminin 5, was also activated in TICs (Fig. 5h) but not in TICs with Col17a1 knockdown (Fig. 5i) or expressing S727A point-mutated STAT3 (Fig. 5j). As expected, FAK was also activated in bulk cells expressing S727E point-mutated STAT3 (Fig. 5k). These data suggest that Col XVII mediates suspension survival through maintaining the stability of laminin 5.

### Col XVII and laminin 5 form hemidesmosome-like structures

To exclude the possibility that suspension survival by TICs in spheres was mediated by merely cell aggregation, we demonstrated the expression of Col XVII and laminin 5 (Supplementary Fig. 4a) and the existence of hemidesmosome-like plaques (Supplementary Fig. 4b), an organized ECM ultrastructure in the basal lamina of the skin epidermis and normal mucosa, in spheres but not in the aggregates of bulk cancer cells formed by hanging drop culture or spheres formed by cancer cells with Col XVII and laminin 5 knockdown (Supplementary Fig. 4b). When subjected to suspension culture for 24 h, the TUNEL staining (Supplementary Fig. 4c) and the levels of cleaved caspase 3 and PARP1 (Supplementary Fig. 4d) were greater in aggregates of bulk cancer cells than in enriched TICs. Moreover, live/dead analysis revealed that all cells in spheres were alive, while only a small number of cells in the centre of the aggregates of bulk cancer cells were alive (Supplementary Fig. 4e). Cells with Col XVII or laminin 5 knockdown decreased in the ability to form spheres, and most of the cells in spheres were dead when subjected to suspension culture (Supplementary Fig. 4e), while showing no change in apoptosis when bulk cancer cells were seeded in monolayer culture (Supplementary Fig. 2d). Moreover, pretreatment of the spheres with enzymes that degrade the components of hemidesmosome, such as chymotrypsin^23^, but not trypsin, an enzyme that does not degrade hemidesmosome, for 5 min, causes cell death in suspension culture (Supplementary Fig. 4e). Together, these data suggest that Col XVII and laminin 5 form an organized structure in spheres, which plays an important role in supporting suspension survival of TICs.

### PP2A serves as an upstream regulator of S727STAT3

**Figure 6:**
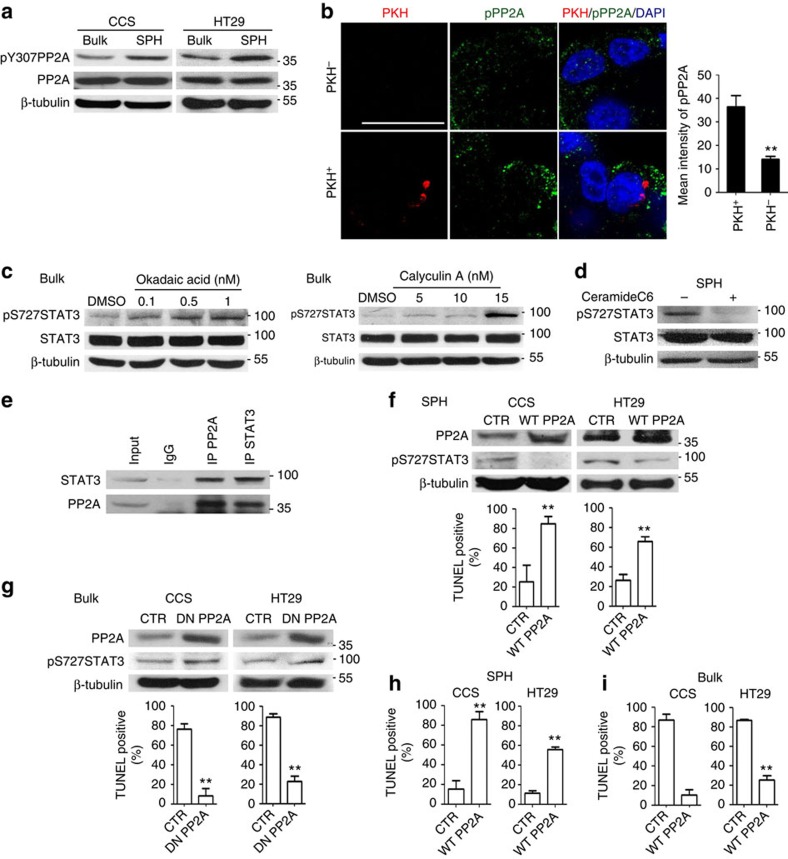
PP2A regulates STAT3 activation at S727 and mediates suspension survival in TICs. (**a**) Bulk cancer cells (Bulk) and spheroid culture-enriched TICs (SPH) were cultured under spheroid condition for 10 h, followed by western blot assay. (**b**) Spheroid culture of PKH-labelled HT29 cells was assayed for immunostaining, and fluorescence intensity was quantified with ZEISS microscope software ZAN. (**c**) Western blot analysis for CCS bulk cancer cells treated with 0, 0.1, 0.5 and 1 nM OA for 1 h or 0, 5, 10 and 15 nM CA for 30 min. (**d**) Western blot analysis for CCS spheroid culture-enriched TICs treated with 50 nM ceramide C6 for 24 h. (**e**) Western blot analysis for immunoprecipitated PP2A or STAT3 complex of CCS bulk cancer cells. (**f**) Spheroid culture-enriched TICs expressing control plasmid (CTR) or WT PP2A were subjected to spheroid condition for 24 h. Top, western blot analysis. Bottom, quantification of TUNEL-positive cells. (**g**) Bulk cancer cells expressing control plasmid (CTR) or DN PP2A were subjected to spheroid condition for 24 h. Top, western blot analysis. Bottom, quantification of TUNEL-positive cells. (**h**) Cells in **f** were subjected to *in vivo* suspension condition for 24 h, followed by TUNEL assay. (**i**) Cells in **g** were subjected to *in vivo* suspension condition for 24 h, followed by TUNEL assay. The results are expressed as mean±s.d. of three independent experiments. Asterisks indicate significant differences (**P<0.01) determined by Student's *t*-test. Scale bar, 20 μm.

For elucidation of the upstream signalling pathways that regulate STAT3 activation at S727, we first showed that enriched TICs did not increase the protein levels of important kinases, such as AKT, P38, JNK and ERK compared with bulk cancer cells (Fig. 3a). However, we found that P38, JNK and ERK are involved in the S727 phosphorylation of STAT3 (Supplementary Fig. 5a). PP2A phosphatase has been reported to inactivate STAT3 via dephosphorylating S727 (refs 24, 25). The phosphorylation of ^Y307^PP2A, which was associated with suppressed PP2A activity^26^, was higher in both enriched TICs (Fig. 6a) and PKH-positive cells in spheroid culture (Fig. 6b). Treatment of bulk cancer cells with PP2A inhibitors, OA^27^ and CA^28^, increased S727 phosphorylation of STAT3 in a dose-dependent manner (Fig. 6c). In contrast, treatment of enriched TICs with PP2A activator, ceramide C6 (ref. 29), reduced S727 phosphorylation of STAT3 (Fig. 6d). Immunoprecipitation assay further showed that PP2A formed a complex with STAT3 (Fig. 6e), suggesting the regulation of STAT3 phosphorylation in TICs by PP2A through a direct interaction. As expected, suspension survival of TICs overexpressing WT PP2A was obviously reduced both *in vitro* and *in vivo* (Fig. 6f,h). In contrast, bulk cancer cells expressing the dominant-negative (DN) form of PP2A increased suspension survival both *in vitro* and *in vivo* (Fig. 6g,i). Consistently, the increase in the PP2A-^S727^STAT3-Col XVII pathway was also demonstrated in TICs enriched by spheroid cultures and ALDH^+^ cells isolated from HCW primary liver metastasis cancer cells, and fresh colorectal cancer specimens (Supplementary Fig. 5b,c).

TICs enriched by spheroid culture from HTB186 brain cancer cells and MCF7 breast cancer cells showed increased expression of Oct4, Nanog and Sox2 (Supplementary Fig. 6a), and increased suspension survival compared with bulk cancer cells (Supplementary Fig. 6b). Moreover, these cells increased in the phosphorylation of PP2A, and subsequent activation of STAT3 at S727 and the expression of Col17a1 (Supplementary Fig. 6c), suggesting that the suspension survival pathway is a general characteristic in enriched TICs derived from a variety of tumours.

### Suspension survival determines TIC properties and prognosis

**Figure 7:**
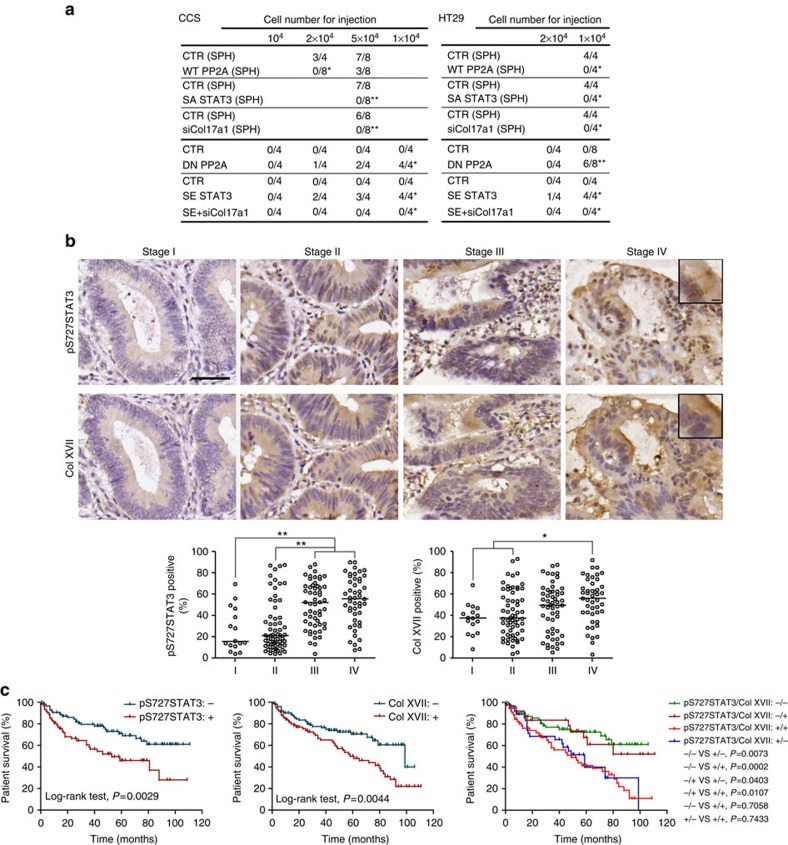
Suspension survival determines tumorigenicity and corresponds to clinical tumour staging and survival. (**a**) Tumorigenicity of bulk cancer cells expressing DN PP2A and S727E point-mutated STAT3 with or without shRNA against Col17a1 or TICs overexpressing WT PP2A, S727A point-mutated STAT3 and shRNA against Col17a1. (**b**) The expression of pS727STAT3 and Col XVII in primary colorectal tumours was detected by immunohistochemical staining of tissue array slides. Top, representative pictures of positive staining of colorectal tumour tissues (different stages) are shown. Bottom, quantification and the correlation between protein expression and tumour stages was subsequently determined. The results are expressed as percentage of diaminobenzidine (DAB)-positive cells, and every single plot represents a patient. The median is shown as a single black line. (**c**) The survival curves of 150 colorectal cancer patients with or without upregulated pS727STAT3 and Col XVII expression (calculated using the Kaplan–Meier method). The expression of pS727STAT3≥40% or Col XVII≥50% was regarded as positive. Asterisks indicate significant differences (*P<0.05, **P<0.01 versus CTR) determined by Fisher's exact test (**a**), one-way ANOVA (**b**) and log-rank test (**c**). Scale bar, 50 μm.

To demonstrate that the increased suspension-survival ability is essential for tumour formation of TICs and has clinical relevance, we first showed that blocking the PP2A-^S727^STAT3-Col XVII pathway inhibited tumour initiation by TICs (Fig. 7a) and activation of the pathway enhanced tumour initiation in bulk tumour cells to the same extent as in TICs (Fig. 7a). We also showed that targeting STAT3 using specific inhibitor, S3I-201, killed TICs in suspension culture (Supplementary Fig. 7a,b) and inhibited tumour initiation (Supplementary Fig. 7c). Interestingly, targeting Col XVII using anti-Col XVII antibody also killed TICs in suspension culture (Supplementary Fig. 7a,b) and inhibited tumour initiation (Supplementary Fig. 7c).

We then examined specimens from 150 colorectal cancer patients and showed an increase in phosphorylation at S727 of STAT3 and Col17a1 expression in correspondence with tumour stages (Fig. 7b), and the expression of these two markers inversely correlated with patient survival (Fig. 7c). Upon performance of multivariate survival analysis, the expression of these two markers inversely correlated with patient survival in stage II+III (*P*=0.046). These data suggest that the suspension survival pathway in TICs has clinical relevance and may be used to develop a new strategy in targeting TICs.

### Suspension survival in TICs determines cancer metastasis

**Figure 8:**
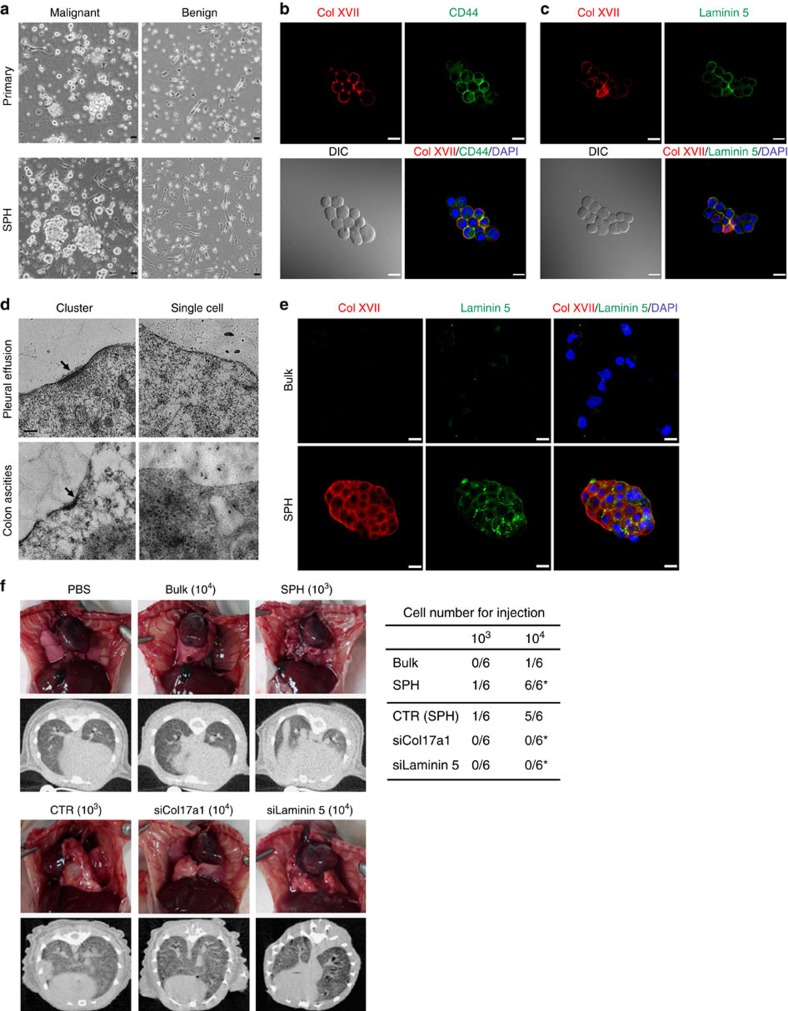
Expression of Col XVII and laminin 5 by TICs in human lung cancer-related malignant pleural effusion and the requirement of the Col XVII/laminin 5 pathway in malignant lung pleural effusion induced by enriched TICs from A549 cells. (**a**) Cells derived from human malignant pleural effusion natively formed spheres, which could be maintained in spheroid culture for weeks, while cells derived from benign pleural effusion of congestive heart failure did not form spheres even when cultured under spheroid conditions. (**b**,**c**) Immunofluorescence revealed that tumour cells in human malignant pleural effusion adopted spheroid morphology and expressed CD44, a surface marker for lung cancer stem cells, Col XVII and laminin 5. (**d**) Cluster fractions and single-cell fractions were separated by passing the pleural effusion fluids or ascites using the cell strainers (with 40-μm pores), followed by transmission electron microscopy (TEM) analysis. TEM images show the existence of hemidesmosomes in the cluster fractions but not in the single-cell fractions of lung cancer malignant plural effusion and colon cancer malignant ascites. Scale bar, 0.1 μm. (**e**) Immunofluorescence revealed the expression of Col XVII and laminin 5 by enriched TIC derived from A549 cells. Scale bar, 20 μm. (**f**) The ability of enriched TICs from A549 cells to form malignant pleural effusion is dependent on the Col XVII-laminin 5 pathway. Micro-CT was performed before killing the mice, to demonstrate intrapleural and extrapulmonary tumour growth. Left, gross images of the pleural cavities of mice receiving injections of indicated numbers of bulk or enriched TICs from A549 cells. Right, the ratio of injections with indicated numbers of cells from different conditions to form malignant pleural effusion. The stem cell frequencies for A549 bulk cancer cells (Bulk) and spheroid culture-enriched TICs (SPH) are 1:25,000 and 1:4,500, respectively. Asterisks indicate significant differences (*P<0.05 versus bulk or CTR) determined by Fisher's exact test.

Malignant pleural effusion caused by lung cancer metastasis is a genuine suspension condition in patients' bodies and contains CD44+ TICs^11^,^30^. After isolation of cancer cells from cytology-confirmed malignant pleural effusion by use of density gradient centrifugation, viable CD44+ cells clustering as spheres were observed and could be maintained in spheroid culture (Fig. 8a,b). However, these clustering cells were not observed in benign pleural effusion (Fig. 8a). Immunofluorescence further showed that these CD44+ cells were also positive for both Col XVII and laminin 5 (Fig. 8b,c). Moreover, transmission electron microscopy revealed that hemidesmosomes existed in cluster cell portions at a density of around two per cell in each 100-nm section (∼200 per cell) but not in single-cell portions of malignant pleural effusion of lung cancers and malignant ascites of colon cancers (Fig. 8d), suggesting that the existence of hemidesmosome structures is restricted to CSCs *in vivo*.

Enriched TICs from A549 (Supplementary Fig. 8a), an anchorage-dependent human lung cancer cell line, increased tumour initiation (Supplementary Fig. 8b), suspension-survival ability (Supplementary Fig. 8c), the PP2A-STAT3-Col XVII-laminin 5 pathway (Supplementary Fig. 8d) and the colocalization of Col XVII and laminin 5 (Fig. 8e). An immunodeficient mouse model of malignant pleural effusion^31^ was then applied to demonstrate that enriched TICs formed malignant pleural effusion as less as 10^3^ cells per injection, while cells in monolayer culture only formed malignant pleural effusion with cell numbers greater than 10^4^ cells per injection (Fig. 8f). Moreover, the ability of enriched TICs to form malignant pleural effusion was significantly blocked by specific short hairpin RNA (shRNA) against Col XVII or laminin 5 but not by control shRNA (Fig. 8f). The relevance of the suspension survival pathway for TICs was further demonstrated using a spontaneous colon cancer metastasis model^32^, where different aliquots of bulk and enriched TICs of HT29 cells were implanted in the caecum, followed by the evaluation of tumour formation and metastasis for up to 16 weeks. Both tumour formation and metastasis rates were increased in mice receiving injection of enriched TICs compared with those receiving injection of bulk HT29 cells (Supplementary Fig. 9). In addition, activation of ^S727^STAT3 induced tumour formation and metastasis of bulk cancer cells, while inactivation of ^S727^STAT3 or knockdown of either Col XVII or laminin 5 reduced both tumour formation and metastasis of enriched TICs (Supplementary Fig. 9). These data together suggest the survival of TICs in a genuine suspension condition and the relevance of the STAT3-Col XVII-laminin 5 pathway for lung cancer malignant pleural effusion and spontaneous colon cancer metastasis.

## Discussion

Efforts for identifying novel characteristics and related signalling pathways of TICs and subsequently determining the targets will lead to the development of new strategies for curing cancer. Here we are the first to identify suspension survival as a characteristic property of TICs. Moreover, we demonstrate that suspension-survival pathway is essential in determining the capacity for sphere formation and tumour initiation in TICs. These findings further reflect a long-believed but not proved theory, ‘seed and soil', proposing the soil, that is, Col XVII and laminin 5-formed organized structure, generated by the seed, TICs themselves, determines their suspension survival. The seed and soil concept was further suggested by means of the malignant lung cancer pleural effusion and colon cancer spontaneous metastasis models, which demonstrated the survival of TICs in genuine suspension conditions and the *de novo* increase in suspension survival by enriched TICs. We finally demonstrated that targeting this pathway blocked suspension survival, sphere formation, tumour initiation and metastasis. Most importantly, we showed the relevance of these findings to clinical patient samples and demonstrated that phosphorylation at S727 of STAT3 and expression of Col XVII could serve as two prognostic factors in colorectal cancer patients.

Although STAT3 has been known to be involved in tumorigenicity and TIC properties^12^,^19^, most of its functions were contributed by Y705 phosphorylation of STAT3. However, there was no difference in phosphorylation levels of STAT3 Y705 between enriched TICs and bulk cancer cells, and the downstream targets of Y705-phosphorylated STAT3 were not upregulated in TICs. More importantly, TICs overexpressed with a DN Y705 point-mutated STAT3 showed no reduction in suspension survival. Contradictory to those of Y705, phosphorylation levels of S727 were increased in enriched TICs compared with bulk cancer cells. Cells expressing an active S727 point-mutated STAT3 increased in suspension survival both *in vitro* and *in vivo*, and showed enhanced tumorigenicity and metastasis, while spheroid culture of cells expressing a DN S727 point-mutated STAT3 failed to survive under suspension conditions and form xenograft tumour and metastasis in mice. S727 phosphorylation has previously been known to activate STAT3, thereby driving prostate tumorigenesis independent of Y705 phosphorylation^33^; this is the first time that S727 of STAT3 was found to play a role in suspension survival, tumorigenicity and metastasis of TICs.

Another novelty of the results is the identification of Col XVII as a putative target of S727-phosphorylated STAT3 and tumorigenicity. Although several downstream targets of Y705-phosphorylated STAT3, such as Mcl-1, Survivin and c-Myc, have been known to mediate the role of Y705-phosphorylated STAT3 in tumorigenicity of prostate cancer^33^, there was no difference in the expression of these mediators between enriched TICs and bulk cancer cells in the current study. In contrast, we identified Col XVII as a downstream target of S727-phosphorylated STAT3 using a genome-wide approach with confirmation of both mRNA and protein levels. The involvement of Col XVII in colon cancer prognosis was supported by a recent study that shows that the expression of Col XVII in 141 cases of colon cancer was significantly associated with a higher tumour, lymph node, metastasis (TNM) stage^34^. In the current study, knockdown of Col17a1 inhibited suspension survival, tumorigenesis and metastasis in TICs, suggesting Col XVII to be a target for cancer therapy. Previously, mutation of Col XVII was only known to play a pathological role in dermal disease, such as epidermolysis bullosa^35^,^36^, where Col XVII participates in keratinocyte adhesion to Col IV, and in p38MAPK-dependent migration and cell signalling^37^. Here we further demonstrated that Col XVII associates with laminin 5 and inhibits the degradation of laminin 5. Both Col XVII and laminin 5 are known to be components of hemidesmosome, and laminin 5 is also known to play a role in mediating suspension survival. Interestingly, we also demonstrate that the ultrastructure of hemidesmosome is present in enriched TICs, and its presence requires both Col XVII and laminin 5. More importantly, we identified the presence of hemidesmosome-like structures in cluster portions rather than single-cell portions of malignant lung cancer pleural effusion and malignant colon cancer specimens. Since TICs form clusters in clinical tumour specimens, such as malignant plural effusion^30^, these data suggest that hemidesmosome-like structures exist in TICs of tumours.

Analysis of the tissue microarray revealed that the expression of pS727STAT3 and Col XVII correlated with tumour staging and inversely with patient survival in colorectal cancer patients. Thus, the activation of this pathway may be a prognostic factor for colorectal cancer patients. Moreover, targeting the STAT3-Col XVII pathway-mediated suspension survival of TICs may help develop new strategies for preventing tumorigenicity and treating cancer. Notably, the percentage of tumour cells positive for Col XVII or pS727STAT3 in our human tumour specimens was high, up to 50% in high-grade tumours. However, the percentage is similar to those of previous reports that used ALDH activity or other stem cell markers to define stem cells^13^. Because there is a lack of reliable markers to identify TICs, the percentages of staining-positive cells may not be equal to the percentages of TICs. However, it is practical to consider that tumours that show increased expression of these putative markers or pathways are those with high stem cell-like signature, the term used by Weinberg *et al.*^38^ in their previous publication. The current manuscript was not aimed to identify the factors that increase the stem cell-like signature in human tumour specimens. Because chemotherapy and anti-angiogenesis have been demonstrated to increase TIC percentage in xenograft or human tumour specimen^39^,^40^, we speculate that tumour therapies, such as chemotherapy, received before tumour resection may result in a high stem cell-like signature in human tumour specimens.

The Hippo-YAP pathway, sensing and responding to the physical organization of cells in tissues, was found to mediate contact inhibition of growth and regulate a variety of cell functions, including quiescence, self-renewal and differentiation of stem cells^41^. Inner cell mass (non-polarized inside cells) and trophectoderm (polarized outside cells) are two different cell lineages existing in mouse embryo at the pre-implantation stage; the former contains pluripotent embryonic stem cells (ESCs) and the latter is a polarized epithelium containing progenitors of an extra-embryonic tissue, placenta. Yap is phosphorylated and cytoplasmic in ESCs, while localizing to the nuclei of trophectoderm and leading to the expression of Cdx2, a differentiated marker of trophectoderm^42^. Progressive multifocal leukoencephalopathy (PML) nuclear bodies (NBs) are essential for the maintenance of ESCs^43^ or haematopoietic stem cells^44^ and TICs of haematopoietic^45^ or solid cancer cells^46^. In glioblastoma stem cells (GSCs), a dramatic increase in the size and number of PML NBs was observed during GSC differentiation^47^. YAP has been known to activate PML transcription^48^; however, nuclear exclusion of YAP by the activated Hippo signalling pathway blocks activation of PML transcription by YAP. These data suggest that YAP is sequestered out of the nucleus by the activated Hippo pathway in stem cells, thereby suppressing the transcription activities of YAP. To show that the Hippo-YAP pathway is affected in TICs in a Col XVII and laminin 5-dependent manner, aggregates of bulk cancer cells and spheres formed in the absence or presence of specific blocking antibodies were subjected to immunofluorescence. We found that bulk cancer cells did not express YAP and PML NBs, despite that some traces of PML were observed in the cytoplasm, while most of the enriched TICs expressed YAP in the cytoplasm and PML NBs in nuclei (Supplementary Fig. 10). Addition of blocking antibodies against Col XVII and laminin 5 induced accumulation of YAP in the nucleus and increased the size and numbers of PML NBs (Supplementary Fig. 10). These data suggest that the Hippo-YAP pathway is affected in TICs via the Col XVII-laminin 5 pathway.

More importantly, our findings are clinically relevant, given that pS727STAT3 and Col XVII are more abundant in late stages and correlate with the survival of colorectal cancer patients, lending support to the notion that the PP2A-^S727^STAT3-Col XVII pathway alone plays an important role in colorectal cancer development, progression and metastasis. We also confirmed that the same pathway was active in TICs of a variety of cancers. More importantly, these findings highlighted the roles of TICs and the Col XVII-laminin 5 pathway in malignant lung cancer pleural effusion and colon cancer metastasis, which may serve as new targets for developing a new strategy in treating cancer, especially for metastasis.

## Methods

### Reagents

Treatment reagents included MG132 (Merk, Schwalbach, Germany), CA (Cell Signaling Technologies, Beverly, MA), OA (Merk), Ceramide C6 (Santa Cruz Biotechnology Inc., Santa Cruz, CA), Cyclohexamide (Merk) and S3I-201 (Merk).

### Primary cells and cell lines

All human materials such as fresh human tumour samples or tissue blocks used in this study were approved by the Institutional Review Board (IRB) of Taipei Veterans General Hospital (Taipei, Taiwan), and singed informed consents were obtained. The CCS and HCW primary cancer cells, provided by Dr Wen K. Yang (China Medical University Hospital, Taichung, Taiwan), were isolated from the primary tumour of a 63-year-old woman with colorectal adenocarcinoma and from a male with liver-metastasised colorectal adenocarcinoma, respectively^12^. HT29 (human colorectal cancer cell line) was obtained from the American Type Culture Collection (ATCC). All of CCS, HCW and HT29 cancer cells were grown in DMEM-HG (Gibco, Grand Island, NY) containing 10% fetal bovine serum (FBS; Gibco). A549 (human lung cancer cell line) was obtained from the ATCC and grown in Ham's F12 (Gibco) containing 10% FBS. MCF7 (human breast cancer cell line) was obtained from the ATCC and grown in DMEM/ F12 (Gibco) containing 10% FBS. HTB186 (human medulloblastoma cell line) was obtained from the ATCC and grown in MEM (Gibco) containing 10% FBS. Primary and cell lines used in the study were confirmed to be mycoplasma-negative before experiments. For spheroid culture, cells were grown in serum-free DMEM-F12 (Gibco) containing 20 ng ml^−1^ epidermal growth factor (EGF) (Peprotech, Rocky Hill, NJ), 10 ng ml^−1^ basic fibroblast growth factor (bFGF) (Peprotech) and N2-supplement (Gibco), with or without PKH26-viable dye labelling (Sigma-Aldrich, St Louis, MO). For PKH26 labelling, cells were labelled with 1:1 mix of 2 × dye for 10 min according to the manufacturer's instructions (Sigma-Aldrich). For *differentiation* culture, the spheres after 15 days of spheroid culture were mixed with growth factor-reduced matrigel (Becton Dickinson, San Jose, CA) and cultured in growth medium with 15% FBS for 1 month. Cells were then recovered by commercial solution (Becton Dickinson) according to the manufacturer's instructions for the subsequent experiments.

### Tissue collection

This study was approved by the Institutional Ethics Committee/IRB of the Taipei Veterans General Hospital. The informed consent was obtained from all subjects. Colorectal cancer specimens with disease grades 3–4 were collected during surgery and immersed in normal saline, brought to the laboratory within 1 h and washed five times with PBS containing 500 U ml^−1^ penicillin and 500 g ml^−1^ streptomycin (Gibco). Samples were minced into small fragments (2 mm) and resuspended in DMEM-HG (Gibco) containing 1 mg ml^−1^ type I collagenase (Sigma-Aldrich) for enzymatic dissociation at 37 °C for 2 h. Released cell samples were subjected to fluorescence-activated cell sorter.

### Flow cytometric analysis and cell separation

Single-cell suspension from spheres was incubated with phycoerythrin-conjugated monoclonal antibodies against human CD133 (catalogue No. 130-080-801; Magnetic cell separation; Miltenyi Biotec Ltd, Surrey, UK) for 30 min at 4 °C, washed twice with PBS and analysed with FACScan flow cytometer (Becton Dickinson). The isolation of cells with high ALDH enzymatic activity was performed by using the ALDEFLUOR kit (StemCell Technologies, Durham, NC) according to the manufacturer's instructions. Briefly, single cells were suspended in ALDEFLUOR assay buffer containing the ALDH substrate and then incubated for 30 min at 37 °C without or with a specific ALDH inhibitor, diethylaminobenzaldehyde as a control. Stained cells were sorted on a FACSAria flow cytometer (Becton Dickinson). Magnetic cell separation of CD133^+^ cells was performed according to the manufacturer's instructions (Auto-Macs; Miltenyi Biotec Ltd). In all experiments, dead cells were gated out using 7-AAD dye.

### Xenograft transplantation and *in vivo* suspension experiment

Study protocols were approved by the Institutional Animal Committee of Taipei Veterans General Hospital. No statistical method was used to determine the sample size. The experiments were not randomized. The male athymic nude mice (BALB/cAnN.Cg-Foxn1nu/CrlNarl, National Laboratory Animal Center) were maintained under specific pathogen-free conditions. The mice used for the experiments were 6–8 weeks of age. Tumour cells were injected subcutaneously and tumour growth was monitored every week for up to 3 months. Animals were excluded only if they died before the pre-determined time point of their killing. For the *in vivo* suspension experiment, aliquots of 10^4^ cells, delivered in 0.5% methylcellulose without or with matrigel (5%), were loaded to DIVVA angioreactors (Trevigen, Gaithersburg, MD) and implanted beneath the skin of nude mice as previously described^49^. After 24 h, cells were recovered from the tubes and processed for TUNEL assay. The investigators were not blinded to allocation during experiments and outcome assessment.

### TUNEL assay

Detection of apoptosis was performed according to the manufacturer's instructions (Roche Molecular Biochemicals, Indianapolis, IN). Cells were fixed with 4% paraformaldehyde, rinsed with PBS, incubated with blocking solution (3% H_2_O_2_ in methanol) for 10 min, rinsed with PBS, permeabilized by 0.1% Triton X-100 in 0.1% sodium citrate for 2 min at 4 °C, incubated with reaction mixture for 60 min at 37 °C in the dark, rinsed with PBS and counterstained with 4,6-diamidino-2-phenylindole (DAPI). Immunofluorescence is observed with fluorescence microscope.

### Annexin V/PI analysis

The early and late apoptotic cells were detected with the Annexin V-FITC Early Apoptosis Detection Kit (Cell Signaling, catalogue No. 6592) according to the manufacturer's instruction. In brief, cells were resuspended in 1 × Annexin V binding buffer, followed by addition of the Annexin V-FITC Conjugate and PI solution. The cells were then incubated for 10 min on ice in the dark, followed by flow cytometry analysis. The data were analysed using the FlowJo software.

### Live/dead analysis

Cell viability was detected using the Nuclear-ID Red/Green cell viability reagent according to the supplier's recommendation (ENZ-53006, Enzo Life Sciences, Farmingdale, NY). The cell viability dye uses a cell-impermeable nucleic acid dye with green fluorescence (excitation/emission: 503/524 nm) to stain dead cells and a cell-permeable nucleic acid dye with red fluorescence (excitation/emission: 568/632 nm) to stain all cells.

### Quantitative real-time PCR

TRIzol reagent (Invitrogen, Carlsbad, CA) was employed to extract total RNA, and RNA was reverse-transcribed using Superscript III RT (Invitrogen) according to the manufacturer's specifications. The quantitative real-time PCR was performed using FastStart SYBR Green Master (Roche Applied Science, Mannheim, Germany) and ABI Step One Real-Time PCR System machine. The sequences of primer sets are listed in Supplementary Table 2.

### Western blot analysis

Cells were lysed and protein was extracted using M-PER (Pierce, Rockford, IL) plus protease inhibitor cocktail (Pierce), with protein concentrations being determined using the BCA assay (Pierce). Aliquots of protein lysates were separated on SDS-10% polyacrylamide gels and transferred on polyvinylidenedifluoride membrane, which was blocked with 5% milk (Bio-Rad, Richmond, CA) in TBST. The membrane was then hybridized with primary antibodies, followed by corresponding secondary antibodies and then detected using a chemiluminescence assay (Millipore, Billerica, MA). Membranes were exposed to an X-ray film to visualize the bands (Amersham Pharmacia Biotech, Piscataway, NJ). Antibodies against pSTAT3 (S727; catalogue No. 9136; 1:1,000), pSTAT3 (Y705; catalogue No. 9131; 1:1,000), STAT3 (catalogue No. 9132; 1:2,000), PP2A C subunit (catalogue No. 4957; 1:2,000), pAkt (S473; catalogue No. 9271; 1:1,000), β-tubulin (catalogue No. 2146; 1:2,000), Flag (catalogue No. 2368; 1:2,000), pAkt (T308; catalogue No. 9275; 1:1,000), Akt (catalogue No. 9272; 1:2,000), ERK (catalogue No. 9102; 1:2,000), pERK (catalogue No. 9101; 1:1,000), pP38 (T180 and Y182; catalogue No. 9211; 1:1,000), P38 (catalogue No. 9212; 1:2,000), pJNK (catalogue No. 9251; 1:1,000) and pmTOR (S2448; catalogue No. 2976; 1:1,000) were purchased from Cell Signaling Technologies. Antibodies against pPP2A (Y307; catalogue No. sc-12615; 1:2,000), Oct4 (catalogue No. sc-9081; 1:500), Nanog (catalogue No. sc-30328; 1:500), Histone H1 (catalogue No. sc-8030; 1:1,000) and Sox2 (catalogue No. sc-20088; 1:500) were purchased from Santa Cruz Biotechnology Inc. Antibodies against pFAK (Y397) (catalogue No. GTX24803; 1:1,000) were purchased from GeneTex (San Antonio, TX). Antibody against Col17a1 (catalogue No. ab28440; 1:500) was purchased from Abcam. Antibody against Laminin γ2 (catalogue No. MAB19562; 1:1,000) was purchased from Chemicon (Temecula, CA). Antibody against cleaved-caspase3 (catalogue No. 1476-1; 1:1,000) was purchased from Epitomics (Burlingame, CA). All the experiments of western blot analysis presented with representative images were repeated at least twice. Uncropped scans of the most important blots are shown in Supplementary Fig. 11.

### Immunoprecipitation

Cell extracts were incubated with antibodies against PP2A (catalogue No. 2038; Cell Signaling Technologies) and STAT3 (catalogue No. 9132; Cell Signaling Technologies) overnight at 4 °C with gentle rotation. The immune complexes were collected by adding protein G beads (Millipore) and were incubated for 1 h at 4 °C with gentle rotation, followed by centrifugation. Precipitates were washed with ice-cold PBS. The precipitates were suspended in SDS sample buffer and analysed using SDS–polyacrylamide gel. Immunoblotting was performed by antibodies against PP2A and STAT3.

### Immunofluorescence

Cells were fixed with 4% paraformaldehyde, reacted with primary antibodies against human CDX2 (Chemicon; catalogue No. AB4123; 1:200), pSTAT3 (S727; catalogue No. 9136; 1:10), cleaved-caspase3 (catalogue No. 1476-1; 1:100), β-tubulin (catalogue No. 2146; 1:10), Col XVII (catalogue No. ab28440; 1:50), Laminin 5 (γ2 chain; catalogue No. MAB19562; 1:50), pPP2A (Y307; catalogue No. sc-12615; 1:50), YAP (catalogue No. 4912; 1:100) and PML (catalogue No. sc-966; 1:100), washed with PBS containing 0.1% Triton X-100, reacted with corresponding DyLight488, DyLight649 or DyLight594-conjugated secondary antibodies (Jackson ImmunoResearch Laboratories, West Grove, PA) and counterstained with DAPI. Immunofluorescence was observed with a fluorescence microscope or a confocal fluorescence microscope. The experiments of immunofluorescence presented with representative images were repeated as indicated in the figure legends.

### Tissue microarray slides and immunohistochemistry

The use of human tissues or tumour specimen samples was approved by the IRB of Taipei Veterans General Hospital. For immunohistochemical staining, paraffin-embedded sections were deparaffinized and rehydrated, with antigen being retrieved by placing sections in Declere working solution (Cell Marque, Austin, TX) in 95 °C water for 30 min. Endogenous peroxidase activity was blocked by 3% hydrogen peroxide. Residual enzymatic activity was removed by washes in PBS, and nonspecific staining was blocked with Ultra V Block for 5 min (Thermo Fisher Scientific, Fremont, CA). Then, the sections were reacted with primary antibodies (pS727STAT3 (catalogue No. 9136; 1:25) and Col17a1 (catalogue No. ab28440; 1:50)), followed by corresponding biotinylated secondary antibodies (Vector Laboratories, Burlingame, CA), and then these sections were treated with streptavidin-peroxidase (LSAB Kit; Dako, Carpinteria, CA), followed by diaminobenzidine staining. Counterstaining was performed with Mayer's haematoxylin and photographed with Zeiss AxioImager Z1 microscope system (Wetzlar, Germany) and an automated acquisition system (TissueGnostics, Vienna, Austria). Pictures were acquired using the Tissue-Faxs software (TissueGnostics). The percentage of positively stained cells was determined using the HistoQuest software (TissueGnostics).

### Plasmid reconstruction

The pHM6-CMV-PP2A (WT and H59K) plasmids were provided by Dr CW Chiang from the Chang-Gung University^50^. The pHM6-CMV-PP2A (WT and H59K) plasmids were subcloned into PCDNA3 plasmids. The pXJ40-CMV-STAT3 (S727A and Y705F) plasmids were obtained from Dr Steven Plelech from Brain Research Center and the Department of Medicine, University of British Columbia^51^. The lentiviral-based expression plasmids for S727A (SA), S727E (SE) and Y705F+S727E (YFSE) point-mutated STAT3 were generated by subcloning pXJ40-CMV-STAT3 (S727A and Y705F) plasmid into pLKO-AS2.puro plasmids, followed by site-directed mutagenesis PCR.

### Lentiviral vector production and cell infection

The shRNA expression plasmids and the bacteria clones for STAT3 (TRCN-00000020840 and TRCN-0000020843) or Col17a1 (TRCN-0000118937 and TRCN-0000118940) were provided by the RNA interference core of the National Science Council in Taiwan. Lentiviral production for shRNA and point-mutated STAT3 overexpression were performed by transfection of 293T cells using Lipofectamine 2000 (LF2000; Invitrogen). Cells were infected in the presence of 8 μg ml^−1^ polybrene (Sigma-Aldrich) and selected with puromycin (1 g ml^−1^).

### Anchorage-independent growth assay

Agarose culture medium (1%) containing 10% FBS was employed to coat the bottom of culture plates. After hardening, 500, 1,000 and 2,500 cells were suspended in agarose culture medium (0.8%) containing 10% FBS and plated onto the bottom layer. Colonies formed in the soft agarose culture were stained with crystal violet and counted 2 weeks after inoculation.

### Gene expression profiling

Total RNA was isolated using the Qiagen RNeasy kit. The generation of labelled cRNA and its hybridization to Human U133 Plus 2 GeneChip arrays (Affymetrix) were carried out at the genomic core facilities in the National Yang-Ming University (Taipei, Taiwan) according to the standard procedures. Functional annotation and identification of over-represented functional themes were performed using the Ingenuity Pathway Analysis web tool developed by Ingenuity Co. All data were deposited in the Gene Expression Omnibus (GEO) database with an accession number of GSE43576.

### ChIP assay

ChIP assay was performed using a commercial kit (Upstate Biotechnology) according to the manufacturer's protocol. HT29 cells with stably overexpressed S727E-mutated STAT3 were fixed with 1% formaldehyde for 20 min at 37 °C and lysed on ice for 10 min in lysis buffer. DNA–protein complexes were sonicated to 200 and 600 base pairs. One aliquot of chromatin was stored for use as input DNA, and the remainder was diluted in immunoprecipitation buffer and incubated overnight (4 °C) with anti-Flag antibody (Sigma-Aldrich). DNA–protein complexes were isolated on salmon sperm DNA/protein A agarose beads and then eluted. Crosslinking was reversed by incubation at 65 °C for 4 h. Proteins were removed with proteinase K. DNA was extracted with phenol/chloroform and PCR-amplified with specific primer for conserved STAT3-binding sequence.

### Luciferase reporter assay

Luciferase assay was performed using the Secrete-Pair Dual Luminescence Assay kit according to the manufacturer's protocol (GeneCopoeia, Rockville, MD). All transfections were performed using nucleofector technology (AMAXA Biosystems, Cologne, Germany) according to the manufacturer's instruction. WT and mutated Col17a1 promoters were cloned in pEZX-PG04 reporter plasmid (GeneCopoeia).

### Ultrastructural analysis

Spheres were fixed in 2.5% glutaraldehyde (phosphate buffer) overnight at 4 °C and postfixed in 2% osmium tetroxide (Millonigs buffer) for 90 min at 4 °C after being washed with phosphate buffer. Specimens were dehydrated through a graded series of ethanol and embedded in Epon 812-equivalent (TAAB Lab). Semithin sections (1 μm) were stained with toluidine blue for light microscopy analysis. Ultrathin sections (40–90 nm) were cut, stained with uranyl acetate and lead citrate and examined with a Hitachi H7600 transmission electron microscope. For pretreatment of spheres with enzymes, spheres were incubated with chymotrypsin (25 ng μl^−1^) and trypsin (25 ng μl^−1^) in medium for 5 min, followed by washing with PBS twice and suspension culture for 24 h.

### Isolation of cancer cells from malignant pleural effusion

Tumour cells from malignant pleural effusion were isolated according to protocols modified from ref. 30. In brief, malignant pleural effusion obtained aseptically in heparinized (10 U ml^−1^) bottles were centrifuged at 200*g* for 20 min at room temperature, and cell pellet was resuspended in 30 ml of cold 1% BSA/2 mM EDTA/PBS. After counting by Trypan Blue exclusion method, cell suspension was layered on Ficoll gradient solution and centrifuged at 800*g* for 30 min at room temperature. After centrifugation, cells contained in the upper gradient were washed once with 1% BSA/2 mM EDTA/PBS and plated in the spheroid medium at a concentration of 10^5^ cells ml^−1^ in ultra-low attachment plates (Corning). Half volume of spheroid medium was added every 3 days.

### Mouse model of malignant pleural effusion

An animal model of malignant pleural effusion was developed by using male athymic nude mice according to the protocols modified from ref. 31. In brief, the animals were anaesthetized with 2% Rompun (Bayer Pharma, Puteaux, France) at 5 mg kg^−1^ and Zoletil 100 (VirbacR, Carros, France) at 30 mg kg^−1^ administered intraperitoneally. Tumour cells were implanted through the chest wall into the left pleural space of mice (intrapleural (i.pl.)) in a volume of 200 μl using a 26-gauge needle. The depth of needle penetration through the intercostal muscles was controlled to avoid lung injury and haemorrhage into the pleural space. Before being returned to their cages, mice were placed until recovery under a heat lamp to maintain body temperature.

### Mouse model of spontaneous colon cancer metastasis

The male athymic nude mice (BALB/cAnN.Cg-Foxn1nu/CrlNarl, National Laboratory Animal Center) used for the experiments were 6 weeks of age. Tumour cells were resuspended in 50 μl HBSS and injected in the caecum through laparotomy, and the weight of the mice was monitored every week for up to 16 weeks. Animals were excluded only if they died before the pre-determined time point for their killing. A complete necropsy procedure was performed at the death of mouse. The tumour initiation was the presence of local tumour at the caecum. The metastasis was the presence of tumour cells in other organs, including the liver, lung and lymph node. Carcinomatosis was defined as the widespread presence of tumour nodules in the peritoneum and bowel serosal surfaces.

### Antibody-blocking assay of spheroid tumour cell

The tumour cells were cultured under spheroid suspension condition for 7 days, followed by treatment with antibodies against Col XVII and laminin 5 or corresponding isotype antibodies for another 3 days. The tumour sphere cells were fixed with 4% paraformaldehyde, followed by frozen-section analysis of the expression of YAP and PML by immunofluorescence.

### Statistical analysis

Comparisons between two groups were analysed by Student's *t*-test. Comparisons within the three groups were analysed by analysis of variance test. Comparison of patient-survival curve was analysed by log-rank test. Comparison of *in vivo* tumorigenicity and metastasis was analysed by Fisher's exact test. A value of *P*<0.05 was considered statistically significant.

### Data availability

The authors declare that the data supporting the findings of this study are available within the article and its Supplementary Information files. Gene expression profile data are deposited in the GEO database with an accession number of GSE43576.

## Additional information

**Accession codes:** Gene sequencing data were deposited in the GEO database with the accession number GSE43576.

**How to cite this article:** Liu, C.-C. *et al.* Suspension survival mediated by PP2A-STAT3-Col XVII determines tumour initiation and metastasis in cancer stem cells. *Nat. Commun.* 7:11798 doi: 10.1038/ncomms11798 (2016).

## Supplementary Material

Supplementary Information Supplementary Figures 1-11 and Supplementary Tables 1-2

## References

[b1] Paget S. The distribution of secondary growths in cancer of the breast. 1889. Cancer Metastasis Rev. 8, 98–101 (1989). 2673568

[b2] Fidler I. J. The pathogenesis of cancer metastasis: the 'seed and soil' hypothesis revisited. Nat. Rev. Cancer 3, 453–458 (2003). 12778135 10.1038/nrc1098

[b3] Duda D. G. *et al.* Malignant cells facilitate lung metastasis by bringing their own soil. Proc. Natl Acad. Sci. USA 107, 21677–21682 (2010). 21098274 10.1073/pnas.1016234107 PMC3003109

[b4] Quintana E. *et al.* Efficient tumour formation by single human melanoma cells. Nature 456, 593–598 (2008). 19052619 10.1038/nature07567 PMC2597380

[b5] Al-Hajj M., Becker M. W., Wicha M., Weissman I. & Clarke M. F. Therapeutic implications of cancer stem cells. Curr. Opin. Genet. Dev. 14, 43–47 (2004). 15108804 10.1016/j.gde.2003.11.007

[b6] Zhou B. B. *et al.* Tumour-initiating cells: challenges and opportunities for anticancer drug discovery. Nat. Rev. Drug Discov. 8, 806–823 (2009). 19794444 10.1038/nrd2137

[b7] Dalerba P. *et al.* Phenotypic characterization of human colorectal cancer stem cells. Proc. Natl Acad. Sci. USA 104, 10158–10163 (2007). 17548814 10.1073/pnas.0703478104 PMC1891215

[b8] Lee J. *et al.* Tumor stem cells derived from glioblastomas cultured in bFGF and EGF more closely mirror the phenotype and genotype of primary tumors than do serum-cultured cell lines. Cancer Cell 9, 391–403 (2006). 16697959 10.1016/j.ccr.2006.03.030

[b9] Weiss S. *et al.* Is there a neural stem cell in the mammalian forebrain? Trends Neurosci. 19, 387–393 (1996). 8873356 10.1016/s0166-2236(96)10035-7

[b10] Reynolds B. A. & Weiss S. Clonal and population analyses demonstrate that an EGF-responsive mammalian embryonic CNS precursor is a stem cell. Dev. Biol. 175, 1–13 (1996). 8608856 10.1006/dbio.1996.0090

[b11] Dontu G. *et al.* *In vitro* propagation and transcriptional profiling of human mammary stem/progenitor cells. Genes Dev. 17, 1253–1270 (2003). 12756227 10.1101/gad.1061803 PMC196056

[b12] Tsai K. S. *et al.* Mesenchymal stem cells promote formation of colorectal tumors in mice. Gastroenterology 141, 1046–1056 (2011). 21699785 10.1053/j.gastro.2011.05.045

[b13] Huang E. H. *et al.* Aldehyde dehydrogenase 1 is a marker for normal and malignant human colonic stem cells (SC) and tracks SC overpopulation during colon tumorigenesis. Cancer Res. 69, 3382–3389 (2009). 19336570 10.1158/0008-5472.CAN-08-4418 PMC2789401

[b14] Chiarugi P. & Giannoni E. Anoikis: a necessary death program for anchorage-dependent cells. Biochem. Pharmacol. 76, 1352–1364 (2008). 18708031 10.1016/j.bcp.2008.07.023

[b15] Reginato M. J. *et al.* Integrins and EGFR coordinately regulate the pro-apoptotic protein Bim to prevent anoikis. Nat. Cell Biol. 5, 733–740 (2003). 12844146 10.1038/ncb1026

[b16] Pece S. *et al.* Biological and molecular heterogeneity of breast cancers correlates with their cancer stem cell content. Cell 140, 62–73 (2010). 20074520 10.1016/j.cell.2009.12.007

[b17] Vachon P. H. *et al.* Differentiation state-selective roles of p38 isoforms in human intestinal epithelial cell anoikis. Gastroenterology 123, 1980–1991 (2002). 12454855 10.1053/gast.2002.37072

[b18] Hsu H. S. *et al.* Mesenchymal stem cells enhance lung cancer initiation through activation of IL-6/JAK2/STAT3 pathway. Lung Cancer 75, 167–177 (2012). 21802163 10.1016/j.lungcan.2011.07.001

[b19] Marotta L. L. *et al.* The JAK2/STAT3 signaling pathway is required for growth of CD44(+)CD24(-) stem cell-like breast cancer cells in human tumors. J. Clin. Invest. 121, 2723–2735 (2011). 21633165 10.1172/JCI44745 PMC3223826

[b20] Zhong Z., Wen Z. & Darnell J. E. Jr. Stat3: a STAT family member activated by tyrosine phosphorylation in response to epidermal growth factor and interleukin-6. Science 264, 95–98 (1994). 8140422 10.1126/science.8140422

[b21] McGrath J. A. *et al.* Mutations in the 180-kD bullous pemphigoid antigen (BPAG2), a hemidesmosomal transmembrane collagen (COL17A1), in generalized atrophic benign epidermolysis bullosa. Nat. Genet. 11, 83–86 (1995). 7550320 10.1038/ng0995-83

[b22] Guess C. M. & Quaranta V. Defining the role of laminin-332 in carcinoma. Matrix Biol. 28, 445–455 (2009). 19686849 10.1016/j.matbio.2009.07.008 PMC2875997

[b23] Douglas W. H., Ripley R. C. & Ellis R. A. Enzymatic digestion of desmosome and hemidesmosome plaques performed on ultrathin sections. J. Cell Biol. 44, 211–214 (1970). 4901375 10.1083/jcb.44.1.211 PMC2107777

[b24] Woetmann A. *et al.* Inhibition of protein phosphatase 2A induces serine/threonine phosphorylation, subcellular redistribution, and functional inhibition of STAT3. Proc. Natl Acad. Sci. USA 96, 10620–10625 (1999). 10485875 10.1073/pnas.96.19.10620 PMC17932

[b25] Liang H. *et al.* Regulation of angiotensin II-induced phosphorylation of STAT3 in vascular smooth muscle cells. J. Biol. Chem. 274, 19846–19851 (1999). 10391929 10.1074/jbc.274.28.19846

[b26] Chen J., Martin B. L. & Brautigan D. L. Regulation of protein serine-threonine phosphatase type-2A by tyrosine phosphorylation. Science 257, 1261–1264 (1992). 1325671 10.1126/science.1325671

[b27] Kamat P. K., Tota S., Saxena G., Shukla R. & Nath C. Okadaic acid (ICV) induced memory impairment in rats: a suitable experimental model to test anti-dementia activity. Brain Res. 1309, 66–74 (2010). 19883632 10.1016/j.brainres.2009.10.064

[b28] Janssens V. & Goris J. Protein phosphatase 2A: a highly regulated family of serine/threonine phosphatases implicated in cell growth and signalling. Biochem. J. 353, 417 (2001). 11171037 10.1042/0264-6021:3530417 PMC1221586

[b29] Chalfant C. E. *et al.* Long chain ceramides activate protein phosphatase-1 and protein phosphatase-2A. Activation is stereospecific and regulated by phosphatidic acid. J. Biol. Chem. 274, 20313–20317 (1999). 10400653 10.1074/jbc.274.29.20313

[b30] Mancini R. *et al.* Spheres derived from lung adenocarcinoma pleural effusions: molecular characterization and tumor engraftment. PLoS ONE 6, e21320 (2011). 21789168 10.1371/journal.pone.0021320 PMC3138755

[b31] Stathopoulos G. T. & Kalomenidis I. Animal models of malignant pleural effusion. Curr. Opin. Pulm. Med. 15, 343–352 (2009). 19387350 10.1097/MCP.0b013e32832af07c

[b32] Tenbaum S. P. *et al.* beta-catenin confers resistance to PI3K and AKT inhibitors and subverts FOXO3a to promote metastasis in colon cancer. Nat. Med. 18, 892–901 (2012). 22610277 10.1038/nm.2772

[b33] Qin H. R. *et al.* Activation of signal transducer and activator of transcription 3 through a phosphomimetic serine 727 promotes prostate tumorigenesis independent of tyrosine 705 phosphorylation. Cancer Res. 68, 7736–7741 (2008). 18829527 10.1158/0008-5472.CAN-08-1125 PMC2859454

[b34] Moilanen J. M. *et al.* Collagen XVII expression correlates with the invasion and metastasis of colorectal cancer. Hum. Pathol. 46, 434–442 (2015). 25623077 10.1016/j.humpath.2014.11.020

[b35] Pasmooij A. M., Pas H. H., Deviaene F. C., Nijenhuis M. & Jonkman M. F. Multiple correcting COL17A1 mutations in patients with revertant mosaicism of epidermolysis bullosa. Am. J. Hum. Genet. 77, 727–740 (2005). 16252234 10.1086/497344 PMC1271383

[b36] Bauer J. W. & Lanschuetzer C. Type XVII collagen gene mutations in junctional epidermolysis bullosa and prospects for gene therapy. Clin. Exp. Dermatol. 28, 53–60 (2003). 12558632 10.1046/j.1365-2230.2003.01192.x

[b37] Qiao H. *et al.* Collagen XVII participates in keratinocyte adhesion to collagen IV, and in p38MAPK-dependent migration and cell signaling. J. Invest. Dermatol. 129, 2288–2295 (2009). 19242520 10.1038/jid.2009.20

[b38] Ben-Porath I. *et al.* An embryonic stem cell-like gene expression signature in poorly differentiated aggressive human tumors. Nat. Genet. 40, 499–507 (2008). 18443585 10.1038/ng.127 PMC2912221

[b39] Deng S. *et al.* Distinct expression levels and patterns of stem cell marker, aldehyde dehydrogenase isoform 1 (ALDH1), in human epithelial cancers. PLoS ONE 5, e10277 (2010). 20422001 10.1371/journal.pone.0010277 PMC2858084

[b40] Lin S. P. *et al.* Colon cancer stem cells resist antiangiogenesis therapy-induced apoptosis. Cancer Lett. 328, 226–234 (2013). 23017941 10.1016/j.canlet.2012.08.036

[b41] Gumbiner B. M. & Kim N. G. The Hippo-YAP signaling pathway and contact inhibition of growth. J. Cell Sci. 127, 709–717 (2014). 24532814 10.1242/jcs.140103 PMC3924201

[b42] Nishioka N. *et al.* The Hippo signaling pathway components Lats and Yap pattern Tead4 activity to distinguish mouse trophectoderm from inner cell mass. Dev. Cell 16, 398–410 (2009). 19289085 10.1016/j.devcel.2009.02.003

[b43] Butler J. T., Hall L. L., Smith K. P. & Lawrence J. B. Changing nuclear landscape and unique PML structures during early epigenetic transitions of human embryonic stem cells. J. Cell Biochem. 107, 609–621 (2009). 19449340 10.1002/jcb.22183 PMC2937361

[b44] Ito K. *et al.* A PML-PPAR-delta pathway for fatty acid oxidation regulates hematopoietic stem cell maintenance. Nat. Med. 18, 1350–1358 (2012). 22902876 10.1038/nm.2882 PMC3566224

[b45] Ito K. *et al.* PML targeting eradicates quiescent leukaemia-initiating cells. Nature 453, 1072–1078 (2008). 18469801 10.1038/nature07016 PMC2712082

[b46] Carracedo A. *et al.* A metabolic prosurvival role for PML in breast cancer. J. Clin. Invest. 122, 3088–3100 (2012). 22886304 10.1172/JCI62129 PMC3433768

[b47] Zhou W. & Bao S. PML-mediated signaling and its role in cancer stem cells. Oncogene 33, 1475–1484 (2014). 23563177 10.1038/onc.2013.111

[b48] Lapi E. *et al.* PML, YAP, and p73 are components of a proapoptotic autoregulatory feedback loop. Mol. Cell 32, 803–814 (2008). 19111660 10.1016/j.molcel.2008.11.019

[b49] Huang W. H. *et al.* Mesenchymal stem cells promote growth and angiogenesis of tumors in mice. Oncogene 32, 4343–4354 (2012). 23085755 10.1038/onc.2012.458

[b50] Chuang E. *et al.* The CD28 and CTLA-4 receptors associate with the serine/threonine phosphatase PP2A. Immunity 13, 313–322 (2000). 11021529 10.1016/s1074-7613(00)00031-5

[b51] Shi X. *et al.* Phosphorylation of STAT3 serine-727 by cyclin-dependent kinase 1 is critical for nocodazole-induced mitotic arrest. Biochemistry 45, 5857–5867 (2006). 16669628 10.1021/bi052490j

