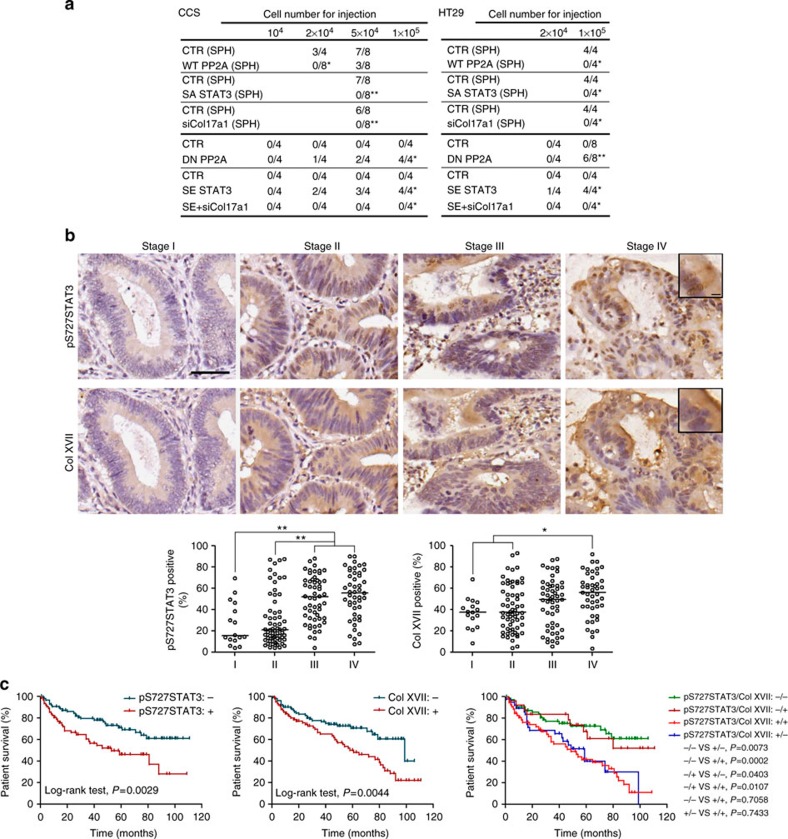# Erratum: Suspension survival mediated by PP2A-STAT3-Col XVII determines tumour initiation and metastasis in cancer stem cells

**DOI:** 10.1038/ncomms14027

**Published:** 2016-12-22

**Authors:** Chen-Chi Liu, Shih-Pei Lin, Han-Shui Hsu, Shung-Haur Yang, Chiu-Hua Lin, Muh-Hwa Yang, Mien-Chie Hung, Shih-Chieh Hung

Nature Communications 7: Article number: 11798; DOI: 10.1038/ncomms11798 (2016); Published 06 16 2016; Updated 12 22 2016

This Article contains errors in Figs 4,6 and 7 that were introduced during the production process. In Fig. 4j, the lane labels on the blot should read in order ‘Input- No Ab-IgG-Flag'. In Fig. 6i, the *x* axis label ‘WT PP2A' of the two graphs should read ‘DN PP2A'. Finally, in Fig. 7a, the maximal cell number reported in the table for the injection of CCS and HT29 should read ‘1 × 10^5^' and not ‘1 × 10^4^'. The correct versions of each figure appear below as Figs 1, 2 and 3, respectively.

## Figures and Tables

**Figure 1:**
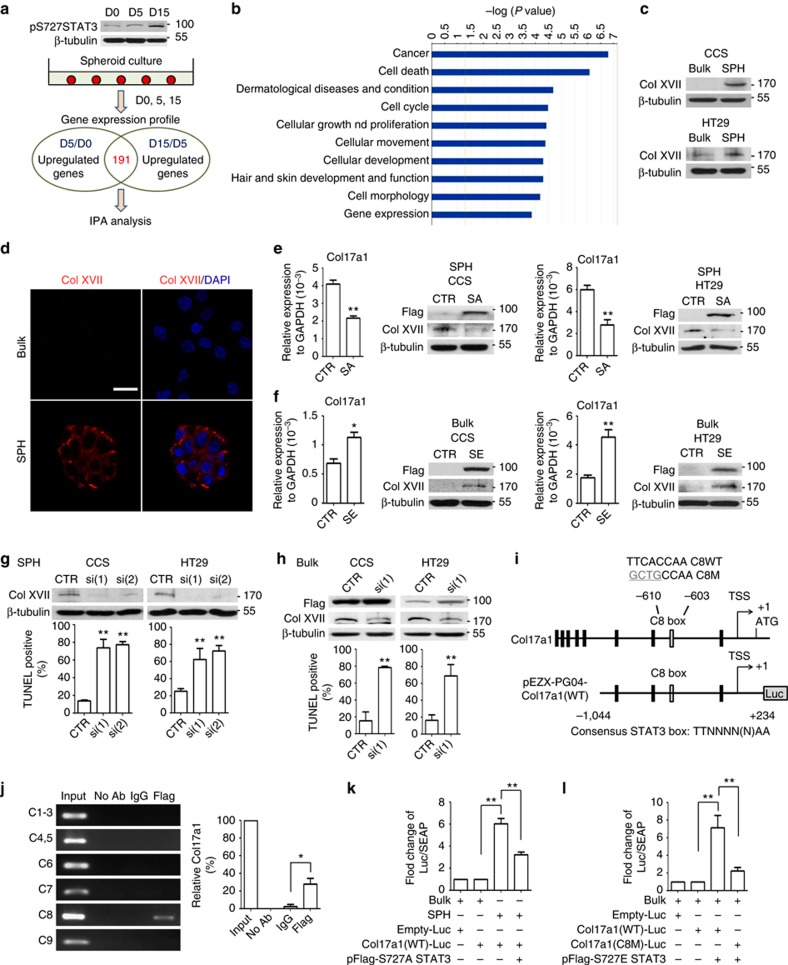


**Figure 2:**
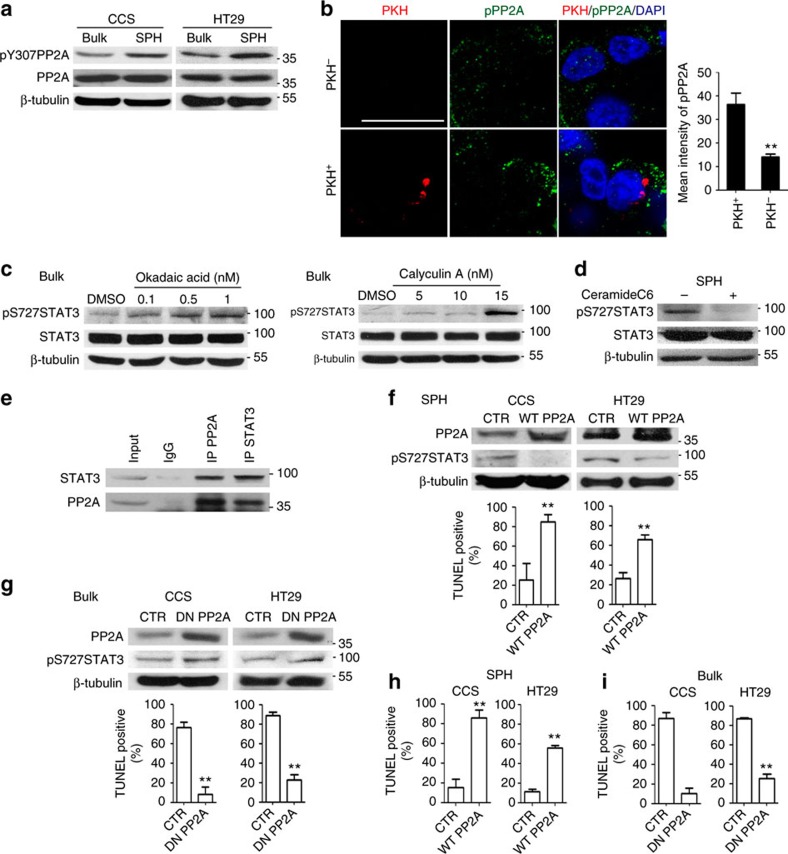


**Figure 3:**