# Supplementary material for: Suspension survival mediated by PP2A-STAT3-Col XVII determines tumour initiation and metastasis in cancer stem cells
Source: Nat Commun. 2016 Jun 16;7:11798. doi: 10.1038/ncomms11798 (PMC4912642; doi:10.1038/ncomms11798)
Supplement: Supplementary Information — Supplementary Figures 1-11 and Supplementary Tables 1-2 [file ncomms11798-s1.pdf]

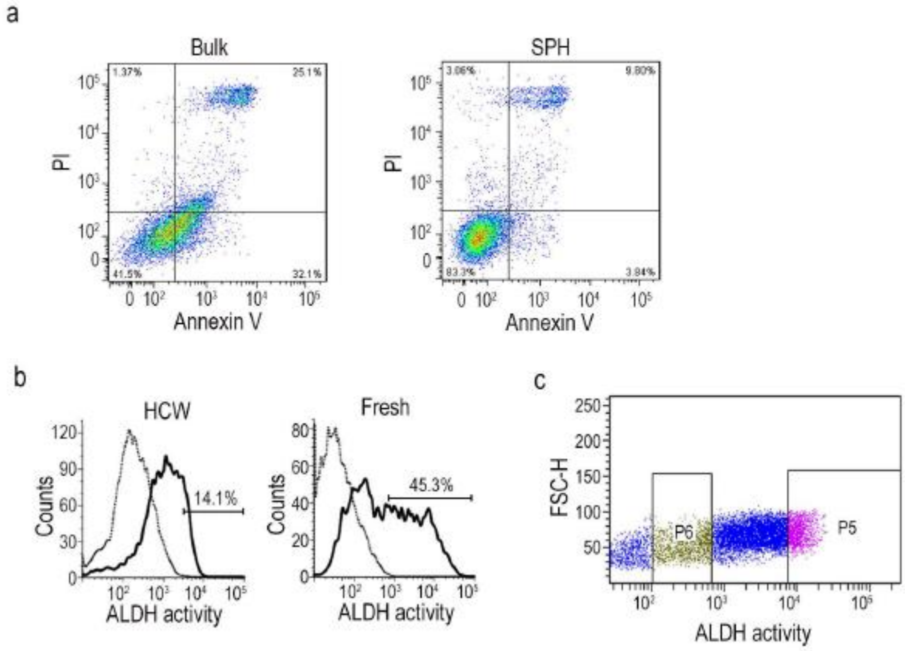

Supplementary Figure 1. Spheroid culture-enriched TICs survive in suspension conditions. (a) Spheroid culture-enriched TICs show better suspension survival ability. HT29 bulk cancer cells (Bulk) and spheroid culture-enriched TICs (SPH) were cultured in spheroid condition for 24 h, followed by analysis of apoptosis and death with Annexin V/PI staining and flow cytometry. The fractions of Annexin V-positive cells for Bulk and SPH were 57.2% and 13.84%, respectively. (b, c) ALDH expression in primary liver metastasis cancer cells. (b) Flow cytometry histogram showing expression of $ALDH^+$ cells in the HCW primary liver metastasis cancer cells and fresh colorectal cancer specimen. (Dotted line, a control with 5 μM of DEAB; solid line, ALDH expression). (c) Methodology of flow cytometry cell sorting in HCW cells. P6, $ALDH^-$ sorting area; P5, $ALDH^+$ sorting area.

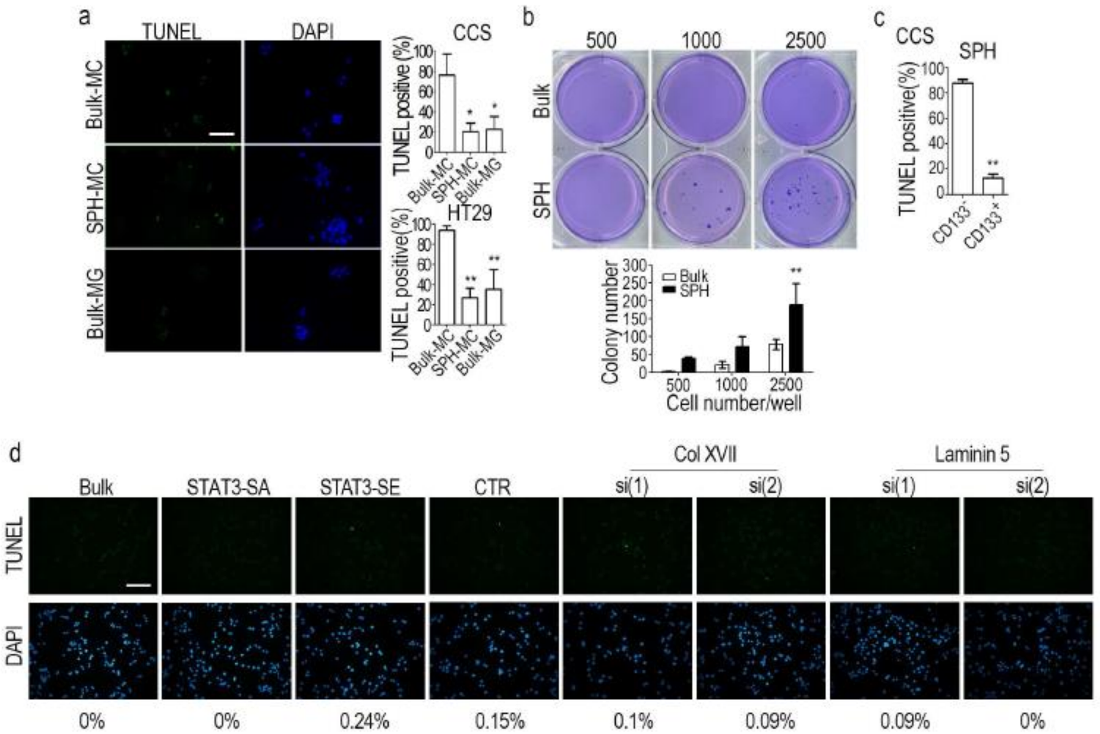

Supplementary Figure 2. Spheroid culture-enriched TICs survive in other suspension conditions. (a) Bulk cancer cells (Bulk) and spheroid culture-enriched TICs (SPH) were cultured in dishes coated with Poly-HEMA (6 mg/ml) containing growth medium and 0.5% methylcellulose (MC) in the absence or presence of 5% matrigel (MG) for 24 h. Left, representative pictures of TUNEL staining for CCS. Bar = 50 μm. Right, quantification of TUNEL positive cell. (b) Top, representative pictures of anchorage-independent growth assay in soft agar for CCS. Bottom, quantification of colonies per well. (c) $CD133^-$ and $CD133^+$ cells isolated from culture-enriched TICs of CCS were cultured in spheroid condition for 24 h, followed by TUNEL assay. (d) The bulk cancer cells expressing S727E and S727A point-mutated STAT3 or with shRNA against Col XVII and laminin 5 were seeded in standard monolayer cell culture for 24 h. The survival abilities were detected by TUNEL assay and the TUNEL positive percentages are shown below the corresponding pictures. The results are expressed as mean ± SD of three independent experiments. Asterisks indicate significant differences (*$P < 0.05$, **$P < 0.01$ versus Bulk) determined by one-way ANOVA (a) and student's t-test (b, c). Bar = 20 μm.

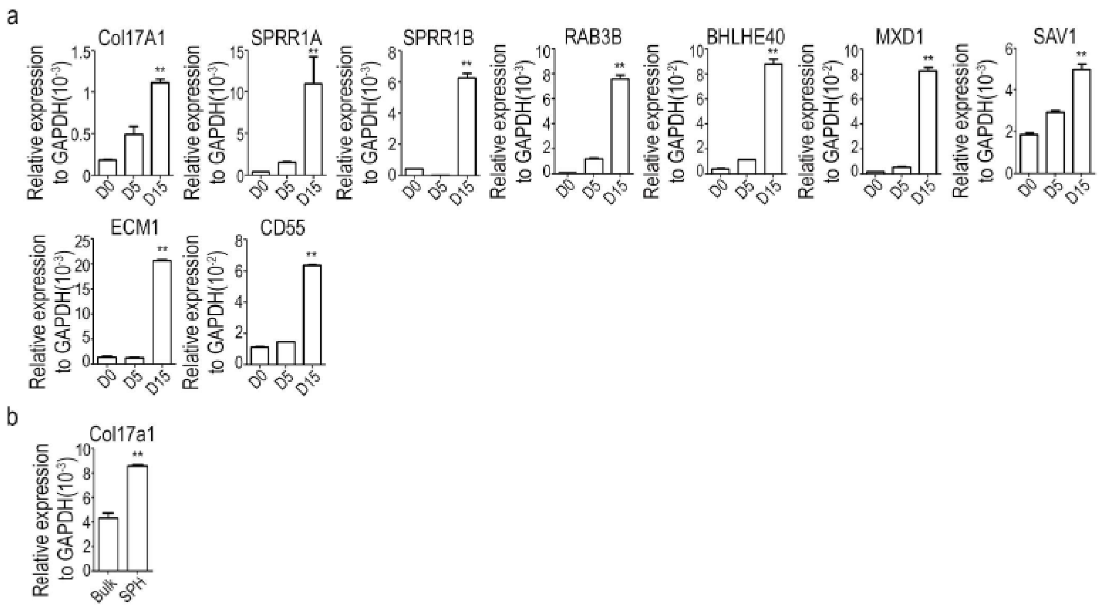

Supplementary Figure 3. Verification of microarray data by quantitative RT-PCR. (a) CCS cancer cells before (day 0), and 5 or 15 days after spheroid culture were harvested for mRNA isolation, followed by quantitative RT-PCR analysis. (b) Quantitative RT-PCR for HT29 bulk cancer cells (Bulk) and spheroid culture-enriched TICs (SPH). Asterisks indicate significant differences (*P < 0.05, **P< 0.01 versus D0 or Bulk) determined by one-way ANOVA (a) and student's t-test (b).

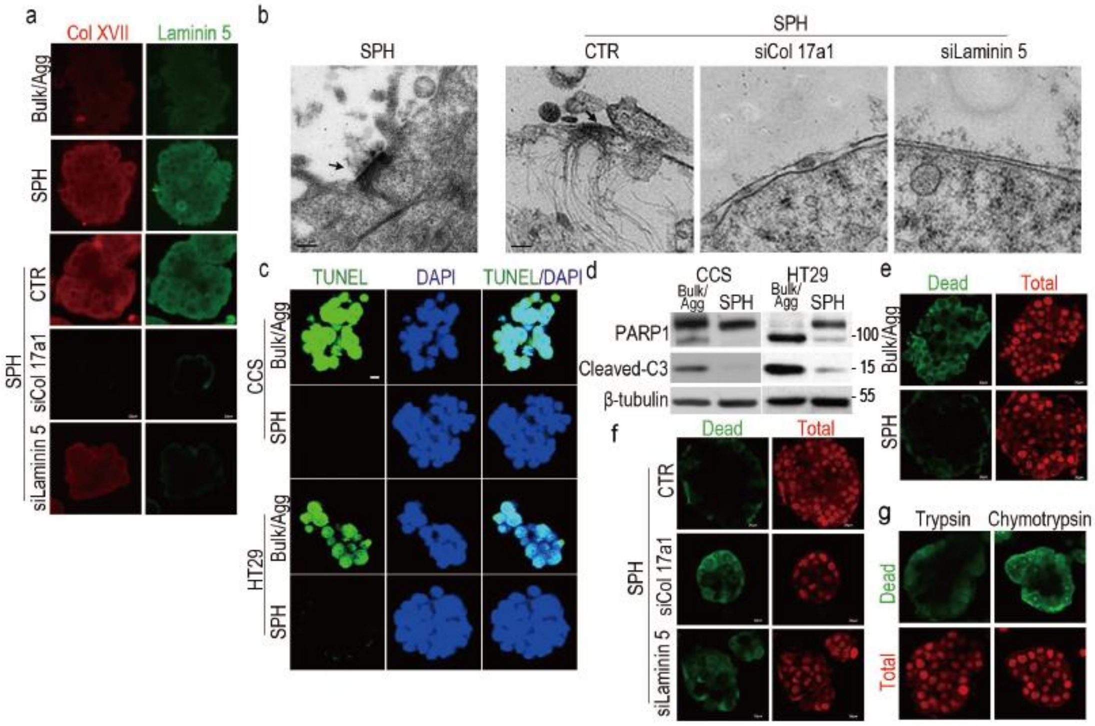

Supplementary Figure 4. Spheroid culture-enriched TICs possess hemidesmosome-like plaques and survive under suspension conditions. (a) Aggregate of HT29 bulk cancer cells formed by hanging drop culture (Bulk/Agg) and sphere formed in HT29 spheroid culture (SPH) without or with shRNA against Col XVII (siCol 17a1) and laminin 5 (siLaminin 5) were subjected to immunofluorescence followed by examination with a confocal microscope. CTR was control scrambled shRNA. Bar = 20 μm. (b) TEM images show the existence of hemidesmosomes in wild-type HT-29 spheroid culture-formed sphere (arrow), but not in cells with shRNA against Col XVII and laminin 5. Bar = 0.2 μm. (c,d) Aggregate of bulk cancer cells formed by hanging drop culture and spheroid culture-enriched TICs were cultured in spheroid condition for 24 h, followed by (c) TUNEL assay and (d) western bolt analysis. Bar = 10 μm. (e, f) Cells in (a) were cultured in spheroid condition for 24 h, followed by Live/Dead analysis. Bar = 20 μm. (g) Spheres formed by spheroid culture treated with trypsin or chymotrypsin treatment for 30 min were subjected to spheroid culture for 24 h followed by Live/Dead analysis. Bar = 20 μm.

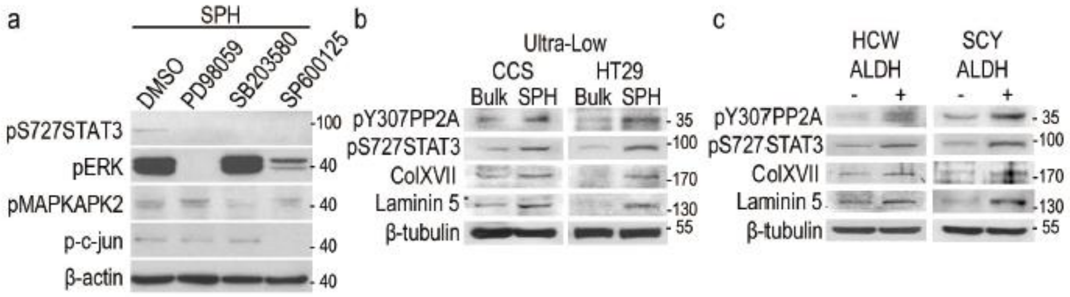

Supplementary Figure 5. Enriched TICs derived from non-adherent culture (Ultra-Low) and ALDH^{+} cancer cells increase in PP2A/ ^{S727}STAT3/ Col XVII pathway. (a) Spheroid culture-enriched TICs (SPH) were cultured in spheroid condition for 10 h in the presence of DMSO (vehicle control), PD98059 (MEK inhibitor), SB203580 (p38MAPK inhibitor) and SP600125 (JNK inhibitor), followed by western blot analysis. (b) Bulk cancer cells (Bulk) and spheroid culture-enriched TICs (SPH) derived from UltraLow culture were cultured in spheroid condition for 10 h, followed by western blot analysis. (c) Western blot analysis for ALDH^{-} and ALDH $^{+}$ cells isolated from HCW primary liver metastasis cancer cells and fresh colorectal cancer specimen.

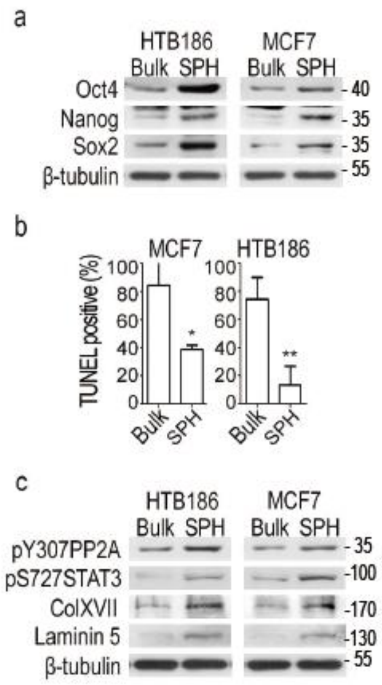

Supplementary Figure 6. Enriched TICs derived from HTB186 and MCF7 cancer cells increase in suspension survival ability and the PP2A/ ^{S727}STAT3/ Col XVII pathway. Bulk cancer cells (Bulk) and spheroid culture-enriched TICs (SPH) were cultured in spheroid condition for (a, c) 10 and (b) 24 h, followed by (a and c) western blot analysis and (b) quantification of TUNEL positive cells. The results are expressed as mean ± SD of three independent experiments. Asterisks indicate significant differences Asterisks indicate significant differences (*P < 0.05, **P< 0.01) determined by student's t-test.

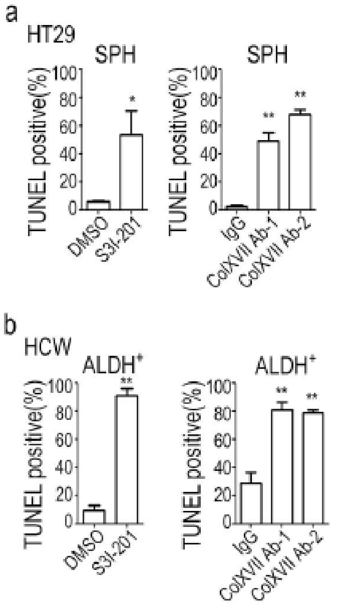

c

HT29	Cell number for injection
	$1x10^5$
DMSO(SPH)	4/8
S3I-201(SPH)	0/8*
DMSO(SPH)-IV	8/8
S3I-201(SPH)-IV	1/8**
IgG	6/8
ColXVII Ab-1	0/8**
ColXVII Ab-2	2/8

Supplementary Figure 7. Targeting the STAT3-Col XVII pathway abrogates suspension survival and tumour initiation after the formation of TICs. (a) Culture-enriched TICs (SPH) of HT29 and (b) $ALDH^+$ cells isolated from HCW primary liver metastasis cancer cells and fresh cancer cells were cultured in spheroid condition for 24 h with treatment of STAT3 inhibitor (S3I-201, 50μM) or 1:25 dilution of the 0.25 mg/ml anti-Col XVII antibody, followed by TUNEL assay. (Ab-1, Abcam; Ab-2, Pierce). The results are expressed as mean ± SD of three independent experiments. (c) Tumourigenicity of enriched TICs (HT29) directly treated with STAT3 inhibitor (S3I-201, 50μM), 1:25 dilution of the 0.25 mg/ml anti-Col XVII antibody or STAT3 inhibitor intravenously (S3I-201, 5mg/kg) post injection of TICs. Asterisks indicate significant differences (*P < 0.05, **P< 0.01 versus DMSO or IgG) determined by student's t-test (two groups in a, b), one-way ANOVA (three groups in a, b), and Fisher's exact test (c).

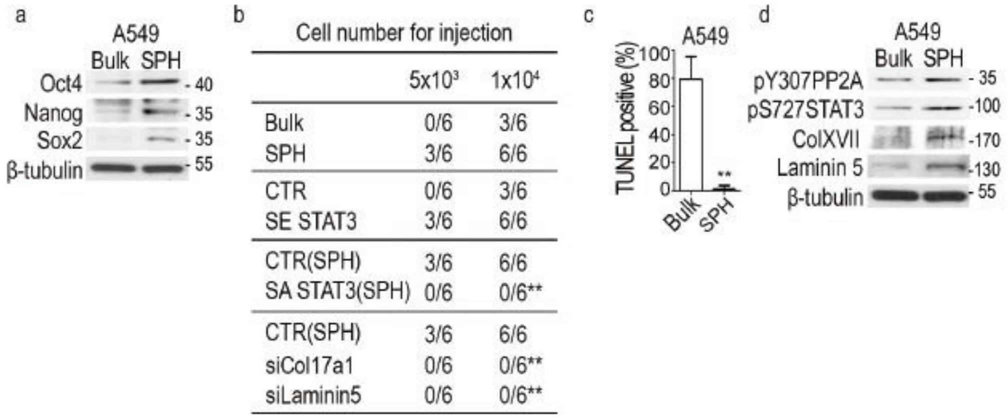

b

Cell number for injection	$5x10^3$	$1x10^4$
Bulk	0/6	3/6
SPH	3/6	6/6
CTR	0/6	3/6
SE STAT3	3/6	6/6
CTR(SPH)	3/6	6/6
SA STAT3(SPH)	0/6	0/6**
CTR(SPH)	3/6	6/6
siCol17a1	0/6	0/6**
siLaminin5	0/6	0/6**

Supplementary Figure 8. Tumourigenicity of bulk cancer cells and spheroid-enriched TICs derived from A549 cells. Bulk cancer cells (Bulk) and spheroid culture-enriched TICs (SPH) were cultured in spheroid condition for (a, d) 10 and (c) 24 h, followed by (a and d) western blot analysis and (c) quantification of TUNEL positive cells. The results are expressed as mean ± SD of three independent experiments. Asterisks indicate significant differences determined by student's t-test. (b) A549 bulk cancer cells and spheroid-enriched TICs without or with genetic modification were subjected to tumourigenicity assay. The stem cell frequencies for bulk cancer cells and TICs are one per 25,000 and 5,000 cells, respectively. Asterisks indicate significant differences (*P < 0.05, **P< 0.01 versus bulk or CTR) determined by Fisher's exact test (b) and student's t-test (c).

Group		Tumor initiation	Metastesis	Dissemination site			
Without genetic modification				liver	lung	L.N.	Carc.
Bulk	10^5 HT29	1/6	1/6	0/6	0/6	1/6	0/6
	10^6 HT29	4/6	3/6	3/6	0/6	3/6	0/6
SPH	10^5 HT29	5/6	3/6	2/6	0/6	3/6	0/6
	10^6 HT29	6/7	5/7	4/7	0/7	5/7	1/7
With genetic modification (10^6 cells)							
Monolayer	CTR	0/6	0/6	0/6	0/6	0/6	0/6
	STAT3 SE	5/10	4/10	0/10	3/10	4/10	1/10
SPH	CTR	5/10	4/10	0/10	2/10	4/10	0/10
	STAT3 SA	2/6	2/6	0/6	0/6	2/6	1/6
	siCol17a1	1/6	1/6	0/6	0/6	1/6	1/6
	siLaminin5	1/6	0/6	0/6	0/6	0/6	0/6

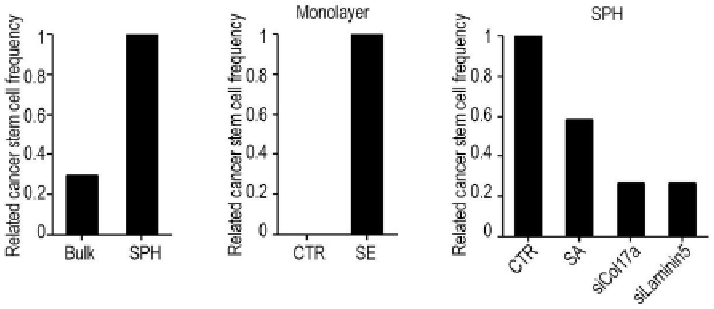

Supplementary Figure 9. The ability of spheroid culture-enriched TICs to develop spontaneous metastasis is dependent on the STAT3-Col XVII-laminin 5 pathway. HT29 bulk cancer cells and spheroid culture-enriched TICs (SPH) without or with genetic modification were injected into the cecum of immunodeficient mice, followed by investigation of orthotopic tumour formation and metastasis up to 16 weeks. Genetic modification included transfection of plasmids carrying S727E point-mutated STAT3 (SE), S727A point-mutated STAT3 (SA), shRNA against Col17a1 and laminin 5. CTR was corresponding control plasmid. Upper, the number of orthotopic tumour formation and metastasis to injection number. Lower, the related ratio of stem cell frequency.

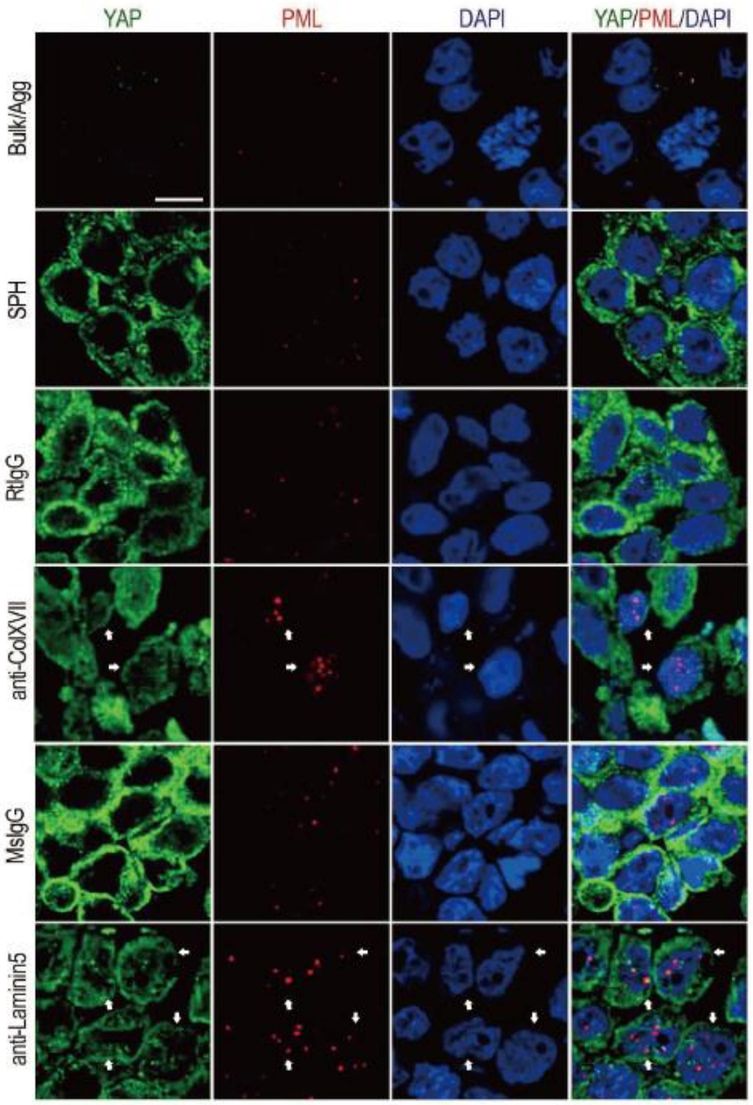

Supplementary Figure 10. The Hippo-YAP pathway is affected in TICs via the Col XVII-laminin 5 pathway. HT29 aggregates of bulk cancer cells formed by hanging drop culture (Bulk/Agg) and spheres formed by spheroid culture (SPH) in the absence or presence of antibodies against Col XVII and laminin 5 or corresponding isotype antibodies were subjected to confocal immunofluorescence analysis for the detection of endogenous YAP and PML. RtIgG, rabbit IgG was the isotype of anti-Col XVII antibody. MsIgG, mouse IgG was the isotype of anti-laminin 5 antibody. Arrows indicate cells that have nuclear Yap. Bar = 10 μm.

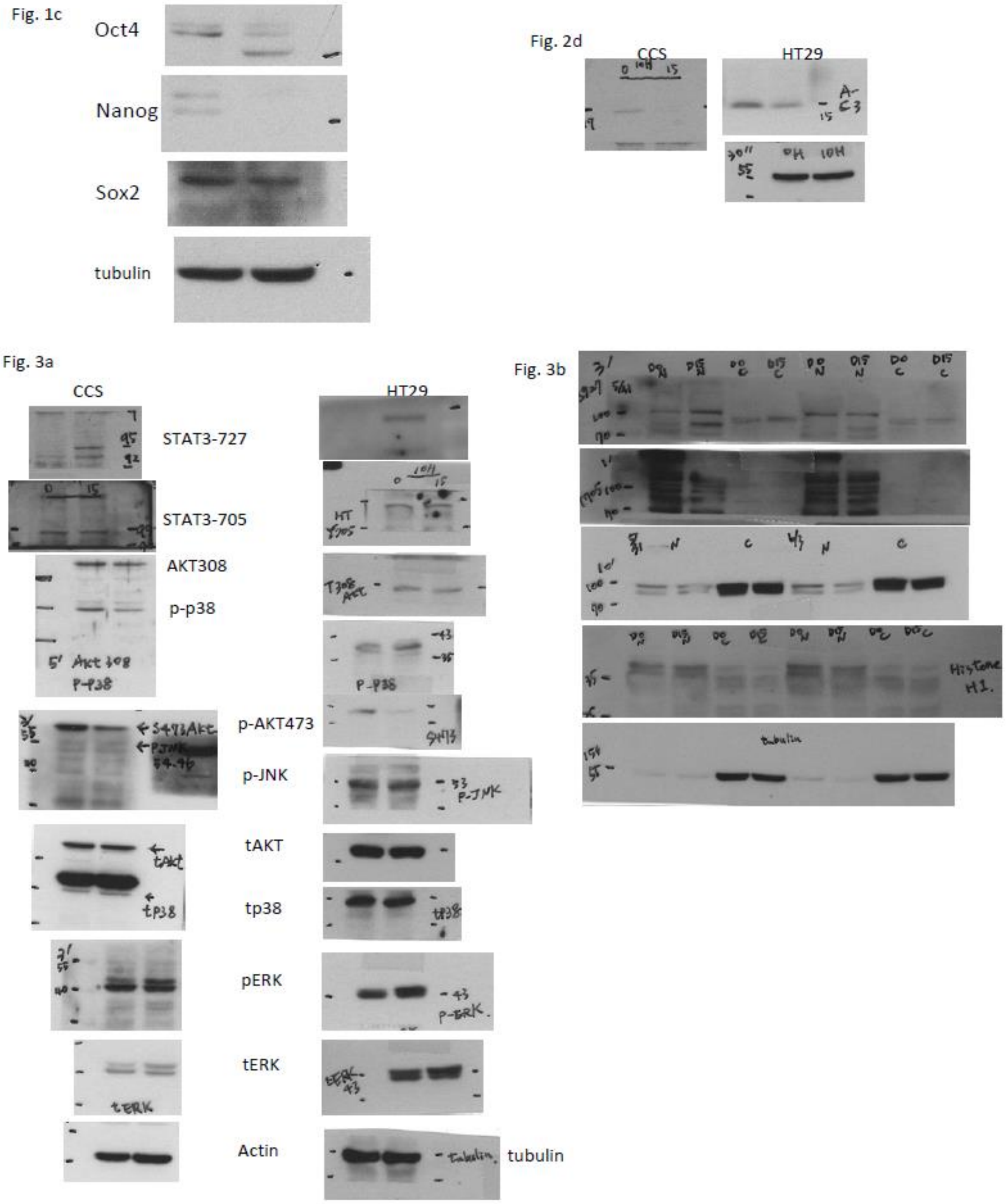

Supplementary Figure 11. Uncropped scans of the most important blots.

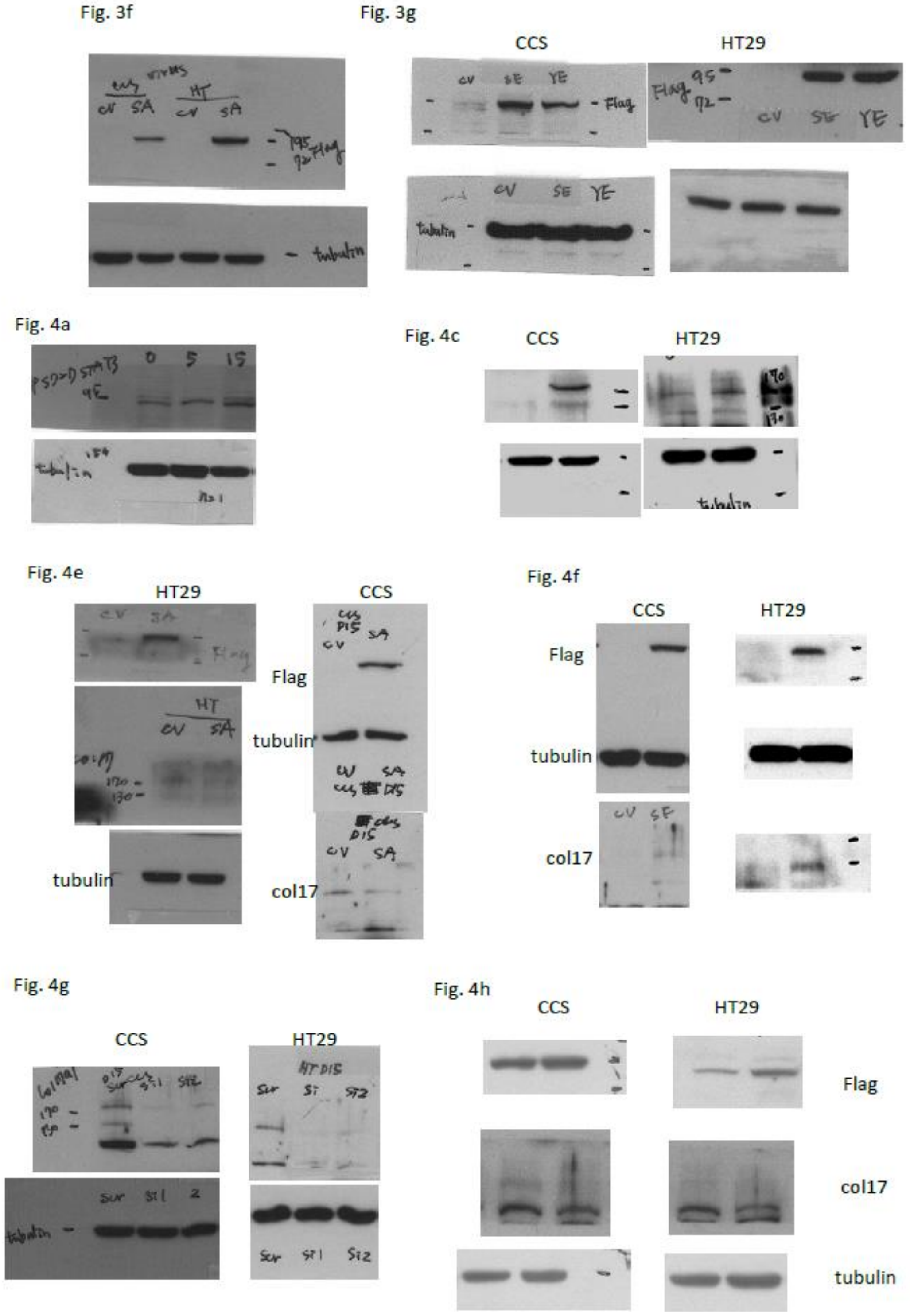

Supplementary Figure 11. Uncropped scans of the most important blots.

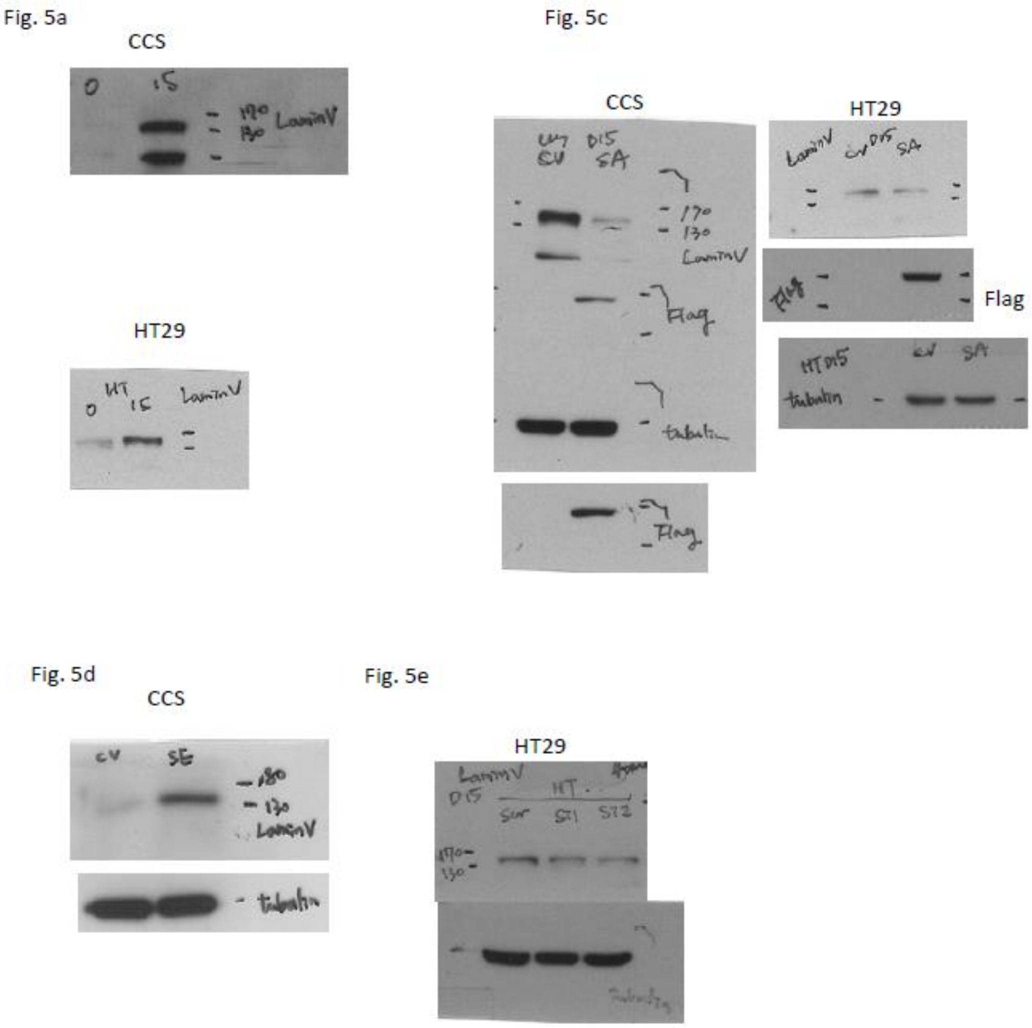

Supplementary Figure 11. Uncropped scans of the most important blots.

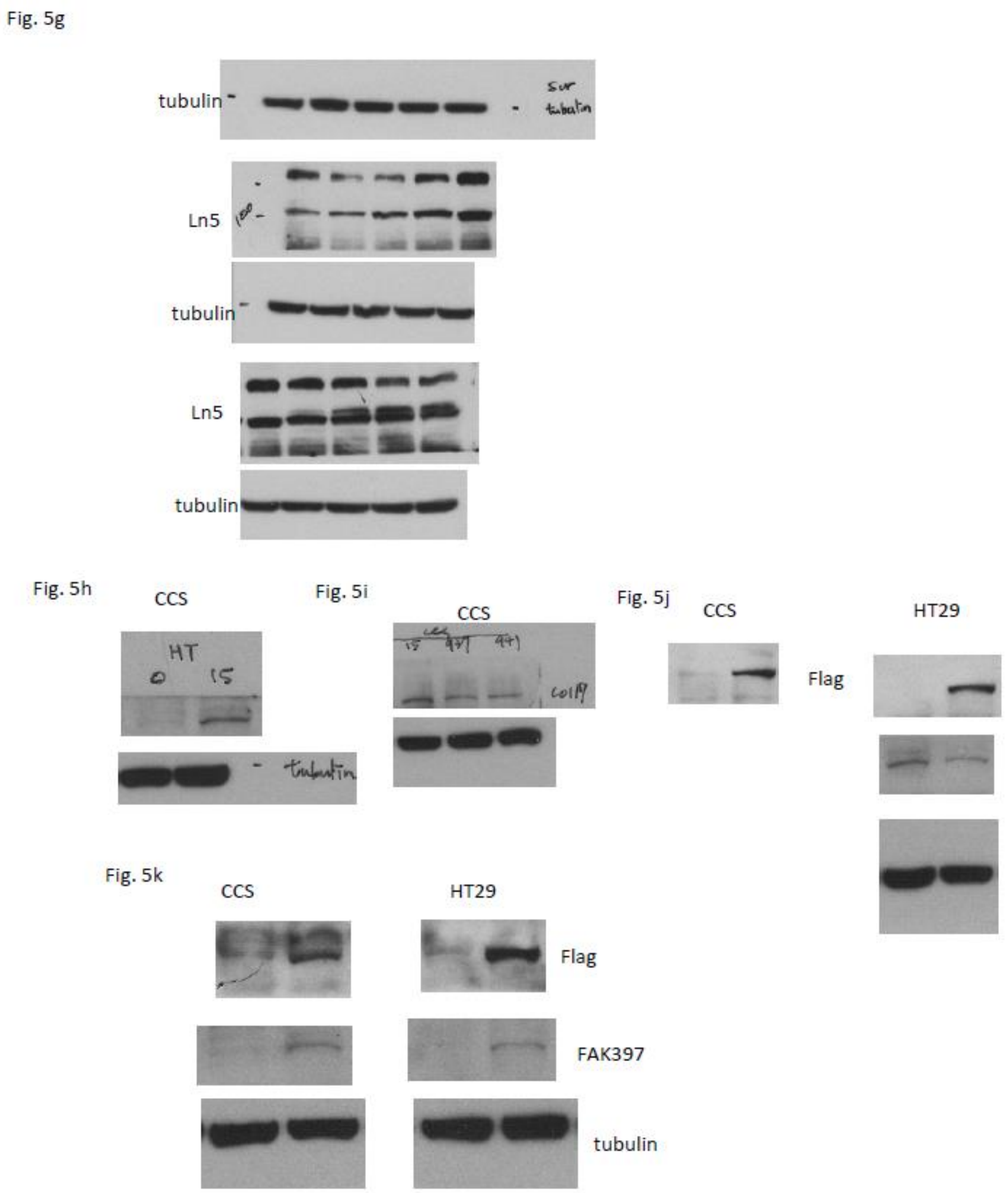

Supplementary Figure 11. Uncropped scans of the most important blots.

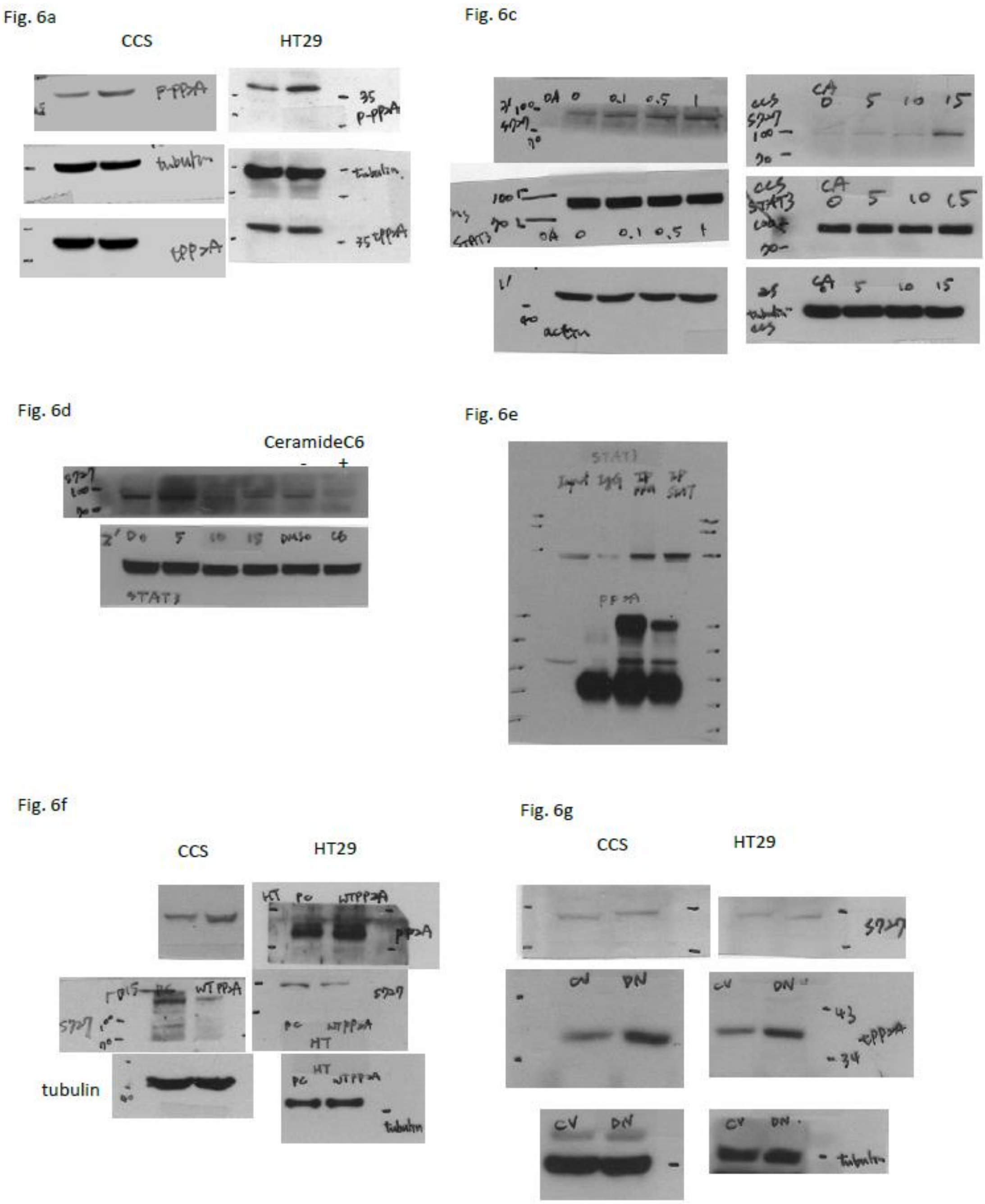

Supplementary Figure 11. Uncropped scans of the most important blots.

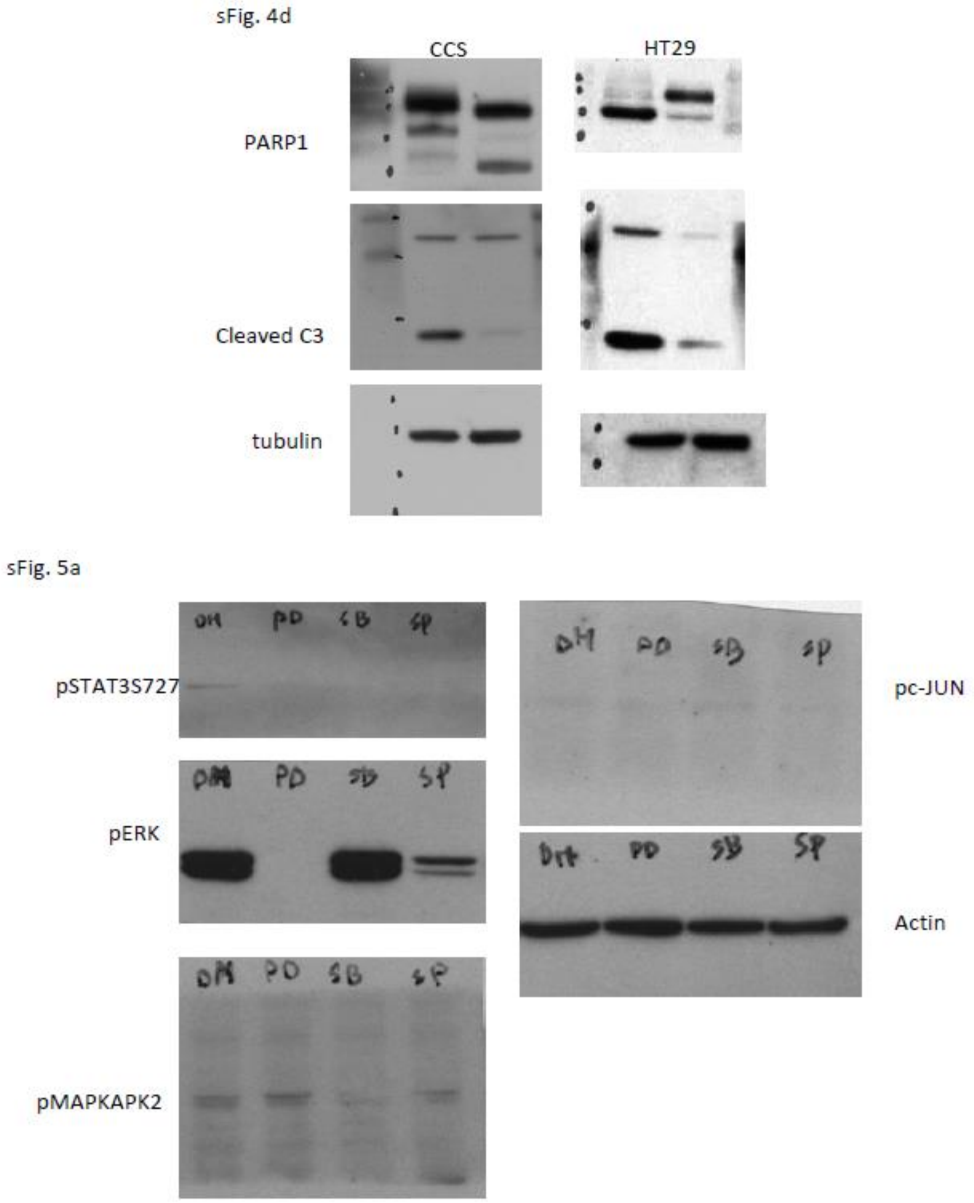

Supplementary Figure 11. Uncropped scans of the most important blots.

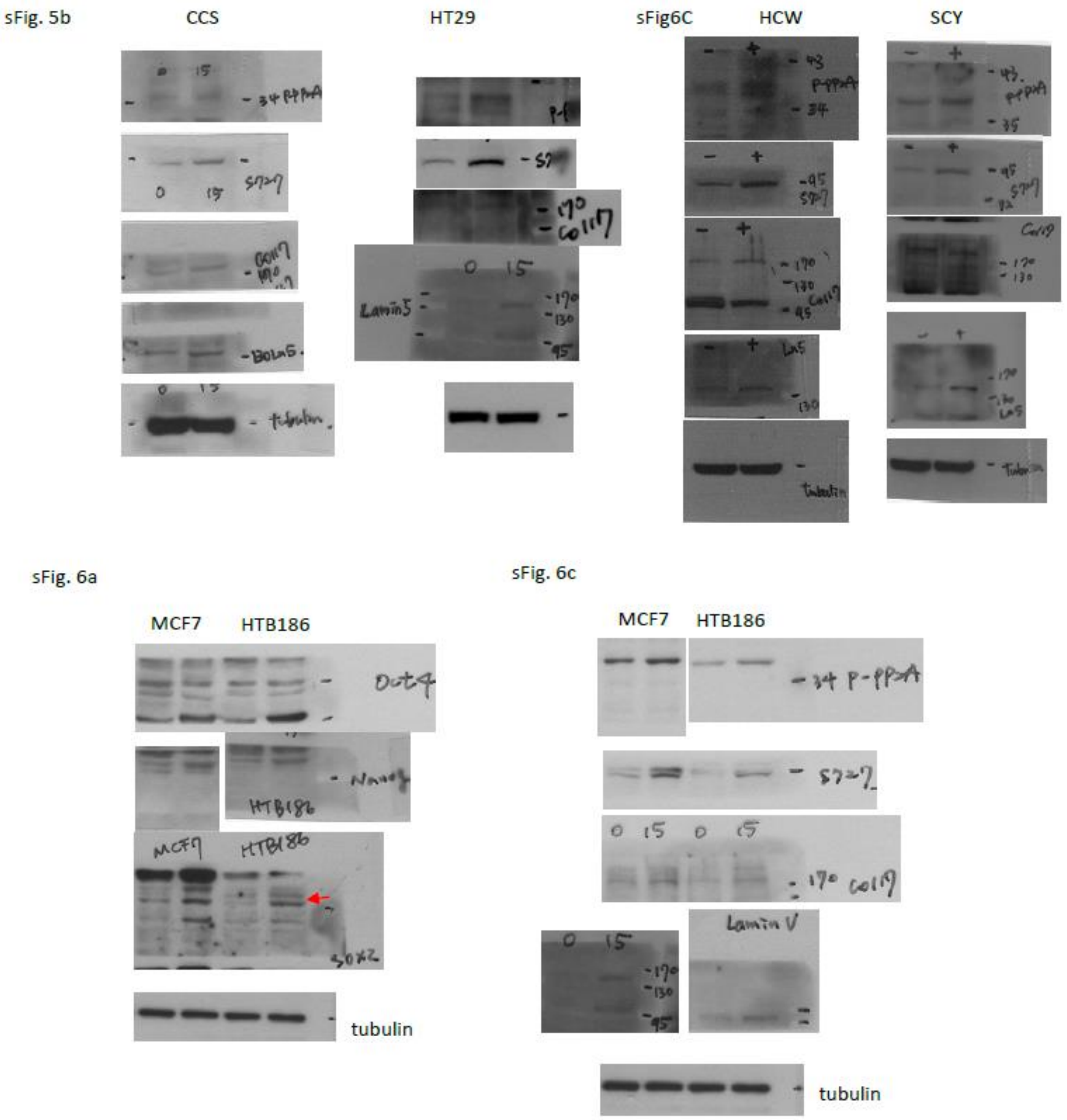

Supplementary Figure 11. Uncropped scans of the most important blots.

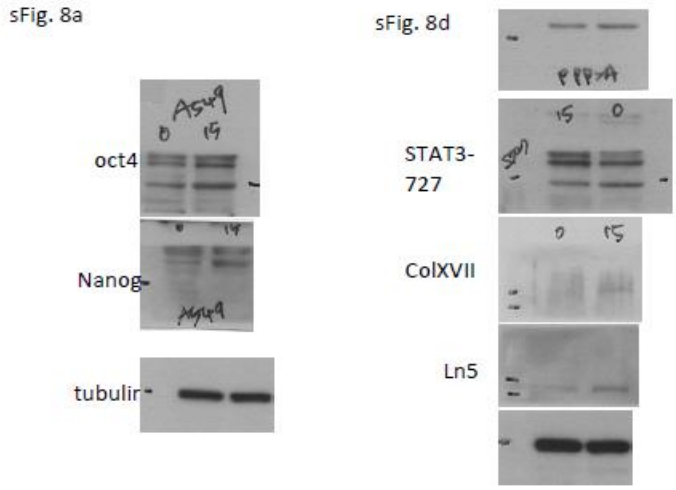

Supplementary Figure 11. Uncropped scans of the most important blots.

Supplementary Table 1. Up-regulated gene expression along with the increase of spheroid culture time

Symbol	Entrez Gene Name	D5/D0	D15/D5	D15/D0(Log2 Ratio)
COL17A1	collagen, type XVII, alpha 1	3.5	2.7	5.5
KRT6B	keratin 6B	3.2	1.7	4.7
CD47	CD47 molecule	4.3	0.8	4.5
KRT15	keratin 15	3.3	1.6	4.5
KRT6A	keratin 6A	2.6	1.9	4.5
SPRR1B	small proline-rich protein 1B	0.6	3.3	4.4
SPRR1A	small proline-rich protein 1A	1.5	2.7	4.1
DHRS9	dehydrogenase/reductase (SDR family) member 9	1.4	2.7	4
ECM1	extracellular matrix protein 1	0.7	3.6	3.6
MXD1	MAX dimerization protein 1	1.6	1.9	3.2
CEACAM1	carcinoembryonic antigen-related cell adhesion molecule 1	2.2	0.9	3.1
HYAL1	hyaluronoglucosaminidase 1	1.6	1.1	3.1
RAB3B	RAB3B, member RAS oncogene family	1.9	0.9	2.9
AQP3	aquaporin 3 (Gill blood group)	1.9	0.9	2.7
CD55	CD55 molecule, decay accelerating factor for complement	1.4	1.6	2.7
SAV1	salvador homolog 1 (Drosophila)	0.8	2.0	2.7
CYP3A5	cytochrome P450, family 3, subfamily A, polypeptide 5	0.7	1.8	2.5
LMO7	LIM domain 7	1	1.3	2.4
STARD4	StAR-related lipid transfer (START) domain containing 4	1.1	1.3	2.4
TMPRSS4	transmembrane protease, serine 4	1.2	1.2	2.4
BHLHE40	basic helix-loop-helix family, member e40	0.7	1.5	2.3
SLC5A3	solute carrier family 5 (sodium/myo-inositol cotransporter)	1	1.7	2.3
LUM	lumican	1.5	1.4	2.2
RASSF6	Ras association (RalGDS/AF-6) domain family member 6	1.2	1.2	2.2

Supplementary Table 2. Primer sequences for real-time PCR

Primer name	Primer sequences
Col17A1 F	5'-AGGCCAGAGCAAACAGAAAA-3'
Col17A1 R	5'-ATGGAGGGTGACGTCTTGAG-3'
SPRR1A F	5'-GACCACACAGCCCATTCTG-3'
SPRR1A R	5'-TAGAGGTGCAAAGGAGCGAT-3'
SPRR1B F	5'-ACTGTTGCAGCATGAGTTCC-3'
SPRR1B R	5'-CTCCTTGGTTTTGGGGATG-3'
ECM1 F	5'-AGCAGCTGGGACTGAGTCAT-3'
ECM1 R	5'-AAGCTTGTCTGGTGGCTGTT-3'
MXD1 F	5'-GTGCCTGGAGAAGTTGAAGG-3'
MXD1 R	5'-CTGAAGCTGGTCGATTTGGT-3'
RAB3B F	5'-GAGAGGGTTGTTCCCACTGA-3'
RAB3B R	5'-AAAGGCCTGCCTTACACTGA-3'
BHLHE40 F	5'-GATCCTGCTGCTTTGCTTTC-3'
BHLHE40 R	5'-CACACACACACACACCCTGA-3'
CD55 F	5'-TTCACCATGATTGGAGAGCA-3'
CD55 R	5'-CTGAACTGTTGGTGGGACCT-3'
SAV1 F	5'-CTGTCCCGAAAGAAAACCAA-3'
SAV1 R	5'-GGCATAAGATTCCGAAGCAG-3'
Laminin V F	5'-GGCTGGTCTTACTGGAGCAG-3'
Laminin V R	5'-CATCAGCCAGAATCCCATCT-3'
GAPDH F	5'-CTCTGCTCCTCCTGTTCGACA-3'
GAPDH R	5'-ACGACCAAATCCGTTGACTC-3'
